# Xylosyl Extension of *O*-Glucose Glycans on the Extracellular Domain of NOTCH1 and NOTCH2 Regulates Notch Cell Surface Trafficking

**DOI:** 10.3390/cells9051220

**Published:** 2020-05-14

**Authors:** Yusuke Urata, Wataru Saiki, Yohei Tsukamoto, Hiroaki Sago, Hideharu Hibi, Tetsuya Okajima, Hideyuki Takeuchi

**Affiliations:** 1 Department of Molecular Biochemistry, Nagoya University Graduate School of Medicine, Nagoya 466-8550, Japan; y-urata.aj@med.nagoya-u.ac.jp (Y.U.); saiki.wataru@e.mbox.nagoya-u.ac.jp (W.S.); tsukamoto.yohei@j.mbox.nagoya-u.ac.jp (Y.T.); sago.hiroaki@a.mbox.nagoya-u.ac.jp (H.S.); 2 Department of Oral and Maxillofacial Surgery, Nagoya University Graduate School of Medicine, Nagoya 466-8550, Japan; hibihi@med.nagoya-u.ac.jp

**Keywords:** xylosyl-extension, *O*-glucosylation, Notch signaling, quality control, xylosyltransferases

## Abstract

Biochemical and genetic studies have indicated that *O*-linked glycosylation such as *O*-glucose (Glc), fucose (Fuc), and *N*-acetylglucosamine (GlcNAc) is critical for Notch signaling; however, it is not fully understood how *O*-glycans regulate the Notch receptor function. Notch receptors are type-I transmembrane proteins with large extracellular domains (ECD), containing 29–36 epidermal growth factor-like (EGF) repeats. Here, we analyzed *O*-Glc glycans on NOTCH1 and NOTCH2 expressed in HEK293T cells using an Orbitrap Fusion mass spectrometer and successfully revealed the structures and stoichiometries of all 17 EGF repeats of NOTCH1 with the *O*-Glc consensus sequence (C1-X-S-X-(P/A)-C2), and 16 out of 17 EGF repeats of NOTCH2 with the same consensus sequence. High levels of *O*-Glc attachment and xylosyl elongation were detected on most NOTCH1 and NOTCH2 EGF repeats. When both glucoside xylosyltransferases, *GXYLT1* and *GXYLT2*, responsible for the xylosyl elongation of *O*-glucose, were genetically deleted, the expression of endogenous NOTCH1 and NOTCH2 on the surface of HEK293T cells did not change, but the cell surface expression of overexpressed NOTCH1 and NOTCH2 decreased compared with that in the wild type cells. In vitro secretion assays consistently showed a reduced secretion of both the NOTCH1 and NOTCH2 ECDs in *GXYLT1* and *GXYLT2* double knockout cells compared with the wild type cells, suggesting a significant role of the elongation of *O*-Glc glycans on the Notch ECDs in the quality control of Notch receptors.

## 1. Introduction

Notch receptors initiate the Notch signaling pathway, an evolutionarily well-conserved pathway essential for development in metazoans [1,2,3,4]. Notch signaling regulates a wide range of cell fate decisions during development as well as stem cell maintenance in adult tissues [5]. Although multiple components are known to regulate the functions of Notch receptors, the glycosylation of Notch receptors has emerged as a critical mechanism for their folding/trafficking, ligand binding, and subsequent signaling [6,7,8,9,10].

Notch receptors function as heterodimers on the surface of cells. They have large extracellular domains (ECD) which contain up to 36 tandemly repeated epidermal growth factor-like (EGF) repeats, and are involved in ligand binding that triggers the activation of the receptors [11]. Although each EGF repeat has a different amino acid sequence, they all show similar folding patterns, where three disulfide bonds via six conserved cysteine residues folded in a specific pattern are needed for Notch receptors to function. The large number of cysteines which must properly pair (216 for 36 EGF repeats) is a huge challenge for the folding of a Notch. Currently, it is not understood how the overall structure of Notch receptors is folded properly and delivered to the cell surface in vivo. The folding of secreted or membrane-bound proteins occurs in the endoplasmic reticulum (ER) [12,13], and the *N*-linked and *O*-linked glycosylation of proteins in the ER play a critical role in the quality control of folding [14,15]. Although the molecular mechanisms by which *N*-linked glycosylation functions in ER quality control are well understood, less is known about how *O*-linked glycosylation on serine or threonine residues of proteins participates in protein folding [16,17].

*O*-Glucose (*O*-Glc) glycans were originally identified on EGF repeats of blood coagulation factors and later on NOTCH1 [18,19]. *O*-Glc modifications were subsequently shown to be essential for proper Notch signaling in *Drosophila* and mice [20,21]. *O*-Glc monosaccharides are added to a serine residue between the first and second cysteine residues of the EGF repeats (C^1^-X-S-X-(P/A)-C^2^). This addition is catalyzed by a single ER-localized protein *O*-glucosyltransferase 1 (POGLUT1, rumi in *Drosophila*) [20,21,22]. Null mutants for *rumi* in *Drosophila* show temperature-sensitive Notch defects with an accumulation of Notch proteins inside the cells, as well as on the surface of cells [20], and mouse mutants in *Poglut1* cause embryonic lethality that phenocopy the Notch mutants [21]. We recently identified POGLUT2 and POGLUT3 that modify sites distinct from POGLUT1 [23]. Glucoside α3-xylosyltransferases (GXYLT1 and GXYLT2, Shams in *Drosophila*) and xyloside α3-xylosyltransferase (XXYLT1 in mammals, CG11388 in *Drosophila*) [24,25] can extend *O*-Glc monosaccharides added by POGLUT1, but not by POGLUT2/3, to form a Xylα1-3Xylα1-3Glc trisaccharide.

We recently found that the cell surface expression of endogenous NOTCH1 in human embryonic kidney (HEK) 293T cells depends on the presence of POFUT1 and POGLUT1 in an additive manner [17]. The results of biochemical and structural experiments indicated that *O*-Fuc and *O*-Glc glycans stabilize EGF repeats via the intramolecular interactions with the underlining polypeptide, without affecting the conformation of the overall structure of the EGF repeat. The mass spectral data of NOTCH1 produced in HEK293T cells showed that the major form of *O*-Fuc glycans are composed of *O-*Fuc monosaccharides, while *O*-Glc glycans are comprised of a trisaccharide at most of the EGF repeats, with an *O*-Glc consensus sequence [26,27]; however, the extent to which *O*-Glc glycans are extended by the xylose residues at each EGF repeat in mammalian Notch receptors remains poorly understood, and whether a xylosyl extension regulates receptor trafficking has yet to be demonstrated.

In the present study, we determined the stoichiometries and structures of *O*-Glc glycans at predicted sites on NOTCH1 and NOTCH2 derived from HEK293T cells. With some exceptions, the majority of the EGF repeats from NOTCH1 and NOTCH2 were modified by the *O*-Glc trisaccharide. Moreover, we found that a xylosyl extension is involved in NOTCH1 and NOTCH2 trafficking in HEK293T cells when expressed at high levels.

## 2. Materials and Methods

### 2.1. Cell Culture

HEK293T cells were purchased from the American Type Culture Collection. HEK293T cells were cultured in a DMEM high-glucose medium supplemented with 10% fetal calf serum (FCS), penicillin, and streptomycin.

### 2.2. CRISPR/Cas9-Mediated Genome Editing of Notch Xylosyltransferases

Gene-specific gRNAs were inserted into a pX330-Cas9-GFP vector, kindly gifted by Dr. Yusuke Maeda and Dr. Taroh Kinoshita [28]. The gRNA sequence for targeting GXYLT1 Exon 1 was 5′-CGCAGCGCATCCCGGCGTGTCGG-3′. The gRNA sequence for targeting GXYLT2 Exon 2 was 5′-GGTGGCCTGTGGCAATCGGCTGCGG-3′. The gRNA sequence for targeting XXYLT1 Exon 1 was 5′-CAGTAGAGCAGGTAACCGGC-3′. The expression vector (5 μg) was transfected in HEK293T cells grown in 10-cm dishes using polyethyleneimine (PEI) max (Cosmo Bio, Tokyo, Japan). Single cell sorting of the Green Fluorescence Protein (GFP)-positive cells was performed in 96-well plates using the FACS SORP Aria2 (BD Biosciences, San Jose, CA, USA) at Nagoya University Graduate School of Medicine. Successful gene editing was confirmed by a genomic PCR with KOD Fx-neo polymerase (TOYOBO, Osaka, Japan) and DNA sequencing.

### 2.3. Expression and Purification of the Extracellular Domain of NOTCH1 and NOTCH2

The plasmid used for the mass spectral analyses were pSecTag2c-mouse NOTCH1 EGF1-36-Myc-His_6_, mouse NOTCH1 EGF1-18-Myc-His_6_, mouse NOTCH1 EGF19-36-Myc-His_6_ or mouse NOTCH1 EGF24-28-Myc-His_6_ [26], and pSecTag2c-mouse NOTCH2 EGF1-36-Myc-His_6_ [23]. HEK293T cells (7.0 × 10^6^) were seeded in a 100/20 mm cell culture dish in DMEM, 10% calf serum, and transiently transfected with 1 μg/well plasmids, using PEI. After 4–5 h, the medium was changed to 6 mL of OPTI-MEM I. The cells were cultured for another 3 days. For the purification of the Notch proteins, the media samples from the transfected cells were applied to Ni-NTA agarose (FUJIFILM Wako Pure Chemical Corporation, Osaka, Japan) affinity chromatography using Poly-prep gravity-flow columns (Bio-Rad, Hercules, CA, USA). After washing with TBS (10 mM Tris buffer pH 8.0 containing 150 mM NaCl) containing 10 mM imidazole, the bound proteins were eluted with TBS containing 250 mM imidazole. The purified samples were analyzed by Western blotting using the anti-Myc antibody (Developmental Studies Hybridoma Bank (DSHB), University of Iowa, Iowa City, IA, USA, 1:2000 dilution) and GelCode blue stain (Thermo Fisher Scientific, Waltham, MA, USA).

### 2.4. Site-Mapping of O-Glycans on the Extracellular Domain of Notch Proteins by Mass Spectrometry

The sample preparation for the mass spectral analysis of the *O*-glucosylation status of the NOTCH1 and NOTCH2 proteins produced in HEK293T cells was performed as previously described, with slight modifications [23]. The purified Notch proteins were reduced/alkylated using 10 mM TCEP (Thermo Fisher) and iodoacetamide, separated on 10% SDS-PAGE, and visualized by GelCode blue staining (Thermo Fisher). The stained bands were excised, followed by in-gel protease digestion using trypsin (Trypsin Gold; Promega, Madison, WI, USA), chymotrypsin (Roche, Basel, Switzerland), or V8 (Glu-C; New England BioLabs, Ipswich, MA, USA). *N*-Glycan cleavage on the glycoproteins was performed using *N*-glycosidase F (Roche). The resulting products were purified and concentrated using Zip-Tip (Thermo Fisher) and injected into Orbitrap Fusion LC-MS (Thermo Fisher) equipped with an Ultimate3000 RSLCnano LC system (Dionex, Thermo Fisher) with a nano HPLC capillary column, 150 mm × 75 μm reverse phase column (Nikkyo Technos, Tokyo, Japan). The (glyco)peptides were separated using a binary gradient solvent system using solvent A (2% acetonitrile and 0.1% formic acid in water) and solvent B (95% acetonitrile and 0.1% formic acid in water) with a flow rate of 300 nl/min. The spectra were recorded with a resolution of 240,000 in the positive polarity mode over the range of *m*/*z* 350–2000 and an automatic gain control target value of 2.0 × 10^5^. The ten most prominent precursor ions obtained in each full scan were isolated for HCD-MS/MS fragmentation with a normalized collision energy of 33 ± 13%, an AGC target of 1.0 × 10^4^, an isolation window of *m*/*z* 3.0, and a dynamic exclusion-enabled fragment resolution of 17,500. The mass spectral data were processed using the Proteome Discoverer v2.3 (Thermo Fisher) and a GlycoPAT software, as previously described [29]. Extracted ion chromatograms (EICs) of all the identified (glyco)peptides were generated using Xcalibur v 4.1 (Thermo Fisher).

### 2.5. Analysis of Endogenous and Overexpressed NOTCH1 and NOTCH2 Expression by Flow Cytometry

The endogenous and overexpressed levels of NOTCH1 and NOTCH2 on the surface of HEK293T cells were analyzed using a CANTO2 flow cytometer (BD BioSciences), as previously described [17]. HEK293T cells were transiently transfected with a pTracer expression vector encoding *N*-terminally FLAG-tagged full length mouse NOTCH1 in which a FLAG tag was inserted into full length Notch1 (pTracer-CMV/Notch1 [30]), a pTracer expression vector encoding *C*-terminally FLAG-tagged full length mouse NOTCH2 [31]), and pMXGFP into the HEK293T cells. After washing the HEK293T cells with HBSS supplemented with 1% BSA, 1 mM CaCl_2_, and 0.02% NaN_3_ (FACS buffer), the cells were incubated with 2 μg/mL PE-conjugated anti-human NOTCH1 (clone: MHN1-519; BioLegend, San Diego, CA, USA), PE-conjugated anti-human NOTCH2 (clone: MHN2-25; BioLegend), or PE-conjugated anti mouse IgG1 (clone: MOPC-21; BioLegend) as a control, 2 μg/mL APC-conjugated anti-DYKDDDDK (clone: L5; BioLegend), APC-conjugated anti-rat IgG2a, κ Isotype Ctrl (clone: RTK2758; BioLegend) as a control, 2 μg/mL APC-conjugated anti-mouse Notch2 (clone: HMN2-35; BioLegend), and APC-conjugated anti-Amenian hamster IgG Isotype Ctrl (clone: HKT888; BioLegend) on ice for 1 h. After washing with 1 mL of FACS buffer twice, the cells then were analyzed with a FACSCantoⅡ (BD Biosciences) flow cytometer. The gate was set to collect the GFP-positive population of 20,000 events for each sample and was analyzed using the FlowJo software (ver. 10.5.3).

### 2.6. Secretion Assay of the Extracellular Domain of NOTCH1 and NOCTH2

The plasmids used for the secretion assays were pSecTag2c-mouse NOTCH1 EGF1-36-Myc-His_6_ or pSecTag2c-mouse NOTCH2 EGF1-36-Myc-His_6_. The empty pSecTag2c vector was used as a negative control. A plasmid encoding human IgG [17] was used as the secretion control. HEK293T cells (5 × 10^5^) were seeded in 6-well dishes in DMEM supplemented with 10% calf serum and transiently transfected with 1 μg/well pSecTag-mouse NOTCH1 EGF1-36-Myc-His_6_ or NOTCH2 EGF1-36-Myc- His_6_ (or empty vector), and 0.05 μg/well IgG plasmid using PEI. After 4–5 h, the medium was changed to 1 mL of OPTI-MEM I. The cells were cultured for another 3 days. Culture media samples were analyzed by a Western blot analysis using the anti-Myc antibody (DSHB, 1: 2000 dilution). The intensity of Notch1 in wild-type cells detected by the Western blot was compared by using the band intensity of IgG to normalize the efficiency of transfection and then calculating the relative value in knockout cells with the wild-type value as 1.

### 2.7. Statistical Analysis

A multiple comparison was performed using Dunnett’s test and Tukey’s test. Dunnett’s test was performed for the results shown in the flow cytometric analysis of endogenous expression of Notch receptors and the secretion assay. Tukey’s test was performed for the results shown in the flow cytometric analysis of overexpressed Notch receptors. *, *p* < 0.05, not significant (n.s.) *p* > 0.05.

## 3. Results

### 3.1. Most EGF Repeats from NOTCH1 and NOTCH2 Are Modified with O-Glc Trisaccharides

**Figure 1:**
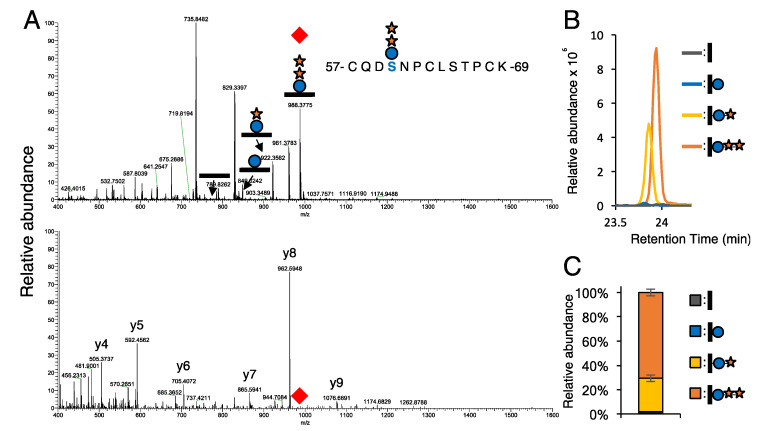
Identification of an *O-*glucosylated peptide from EGF2 from NOTCH1 by LC-MS/MS. NOTCH1 EGF1-18 protein was produced in the wild type HEK293T cells and purified from the culture medium, as described in the Materials and Methods. The protein was reduced, alkylated, and purified by SDS-PAGE, and subjected to in-gel trypsin digestion. The resulting peptides were analyzed by LC-MS/MS, as described in the Materials and Methods. (**A**) The top panel show an MS spectrum of 23.8 to 24.0 min. The bottom panel shows the MS/MS spectrum of the glycopeptide from EGF2 modified with a Xyl-Xyl-Glc trisaccharide. The red diamond denotes the parental ion. Numerous b- and y-ions confirmed the identity of the peptide 57-CQDSNPCLSTPCK-69 from NOTCH1. (**B**) Extracted ion chromatograms (EICs) searched for the glycopeptides modified with different glycoforms are shown. (**C**) Quantification was performed using the height of the EICs of the detected ions and the total amount of detected (glyco)peptides with different glycoforms set at 100% (*n* = 3). Error bar shows standard error of the mean. Black bar—naked peptide; blue circle—glucose; orange star—xylose.

**Figure 2:**
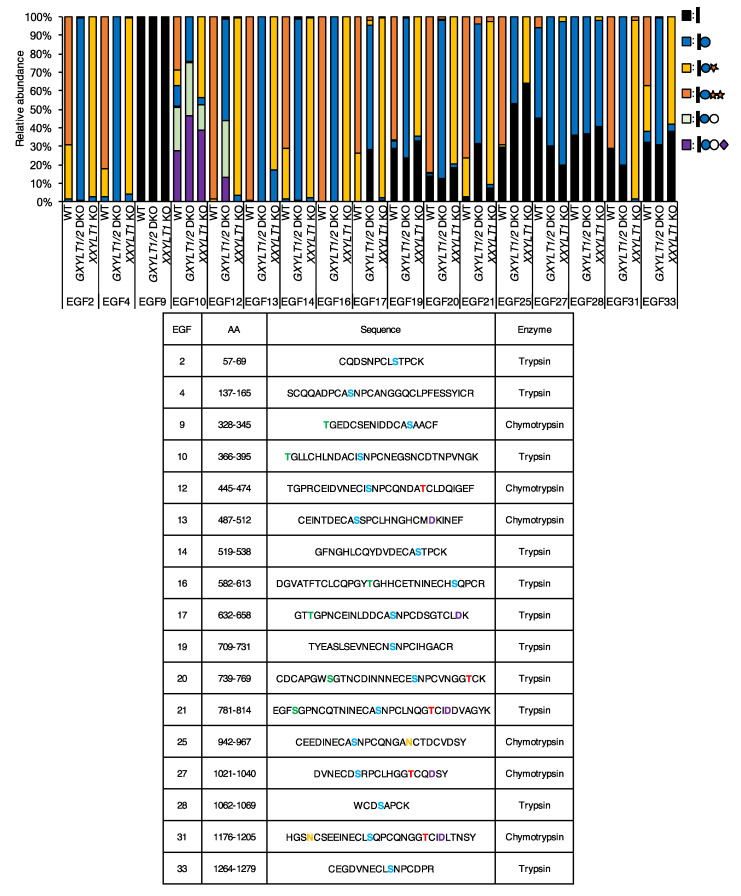
Epidermal growth factor-like (EGF) repeats from NOTCH1 are modified with *O*-Glc trisaccharides. Mass spectrometric analysis of *O*-Glc glycans on mouse NOTCH1 produced in wild type control cells, *GXYLT1/2* double knockout (DKO) cells, and *XXYLT1* knockout (KO) cells. Samples were generated in wild type control HEK293T cells, *GXYLT1/2* DKO cells, and *XXYLT1* KO cells transfected with the plasmids encoding the mouse NOTCH1 ECDs as described in Experimental Procedures. The data are derived from the analysis of mouse NOTCH1 EGF1-18, mouse NOTCH1 EGF19-36, and mouse NOTCH1 EGF24-28. MS/MS spectra confirmed the identity of (glyco)peptides based on the presence of peptide-specific b- and y-ions and the neutral loss of the predicted glycans. Spectra of MS/MS are shown in Figure S1. The sequence of peptides, the predicted and measured mass (*m*/*z*), and the charge state are summarized in Table S1. Quantification was performed using the height of the EICs and the total amount of detected (glyco)peptides with different glycoforms is set at 100%. Data are derived from at least two biological replicates. Further details are shown in Table S1. Colored letters in the sequences of the table show the predicted post-translational modification sites. Blue—*O*-Glc; red—*O*-Fuc; green—*O*-GlcNAc; yellow—*N*-glycan; purple—β-hydroxylation.

**Figure 3:**
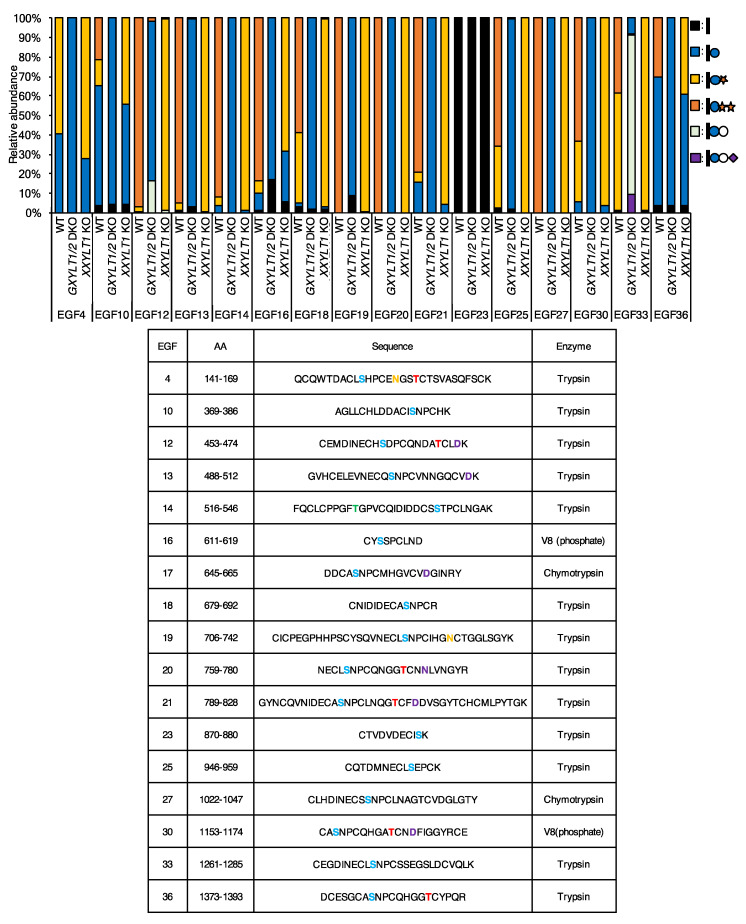
EGF repeats from NOTCH2 are modified with *O*-Glc trisaccharides. Mass spectrometric analysis of *O*-Glc glycans on mouse NOTCH2 produced in HEK293T cells. Samples were generated in wild type control HEK293T cells, *GXYLT1/2* DKO cells, and *XXYLT1* KO cells transfected with the plasmids encoding mouse NOTCH2 extracellular domains (ECDs) as described in Experimental Procedures. The data are derived from the analysis of mouse NOTCH2 EGF1-36. MS/MS spectra confirmed the identity of (glyco)peptides based on the presence of peptide-specific b- and y-ions and the neutral loss of the predicted glycans. Spectra of MS/MS are shown in Figure S2. The sequence of peptides, the predicted and measured mass (*m*/*z*), and the charge state are summarized in Table S2. Quantification was performed using the height of the EICs and the total amount of detected (glyco)peptides with different glycoforms is set at 100%. Data are derived from at least two biological replicates. Further details are shown in Table S2. Colored letters in the sequences of the table show the predicted post-translational modification sites. Blue—*O*-Glc; red—*O*-Fuc; green—*O*-GlcNAc; yellow—*N*-glycan; purple—β-hydroxylation.

In order to identify the *O*-Glc glycoforms that exist on each EGF repeat on NOTCH1 or NOTCH2, we transfected the expression plasmids encoding EGF repeat 1 (EGF1) up to EGF36 of mouse NOTCH1 and NOTCH2 with *C*-terminal MycHis_6_ tags into HEK293Tcells. The secreted Notch proteins were purified from the culture media by Ni-NTA affinity chromatography, reduced and alkylated, and subjected to digestion with proteases, followed by a mass spectrometry analysis. Depending on the site of modification, different levels of sensitivity were observed. Some EGF repeats, such as EGF2 from NOTCH1, were readily detected (Figure 1, Figure S1, Table S1), while others were not. The selection of specific proteases and buffers and the optimization of modes with a single level of collision energy (CE) or with mixed CEs during the mass spectral analysis helped us accomplish the semi-quantitative characterization of *O*-Glc glycoforms on almost all of the EGF repeats from NOTCH1 and NOTCH2 (Figure 2 and Figure 3; Supplementary Materials, Figures S1 and S2, Tables S1 and S2). For example, for the detection of *O*-Glc glycans on EGF16 and EGF30 of NOTCH2, we took advantage of the specificity of the Glu-C (V8) protease, which cleaves at the *C*-terminus of aspartic acid residues more efficiently in phosphate buffers than in other buffers, such as ammonium bicarbonate and ammonium acetate.

NOTCH1 analysis revealed that 70% or more of the *O*-Glc glycoforms on 12 out of 17 EGF repeats were xylose-extended *O*-Glc glycans (Figure 2, Figure S1, Table S1). As for the other EGF repeats, 50–70% of EGF25, EGF27, and EGF28 were modified with *O*-Glc glycans. The majority of the *O*-Glc glycoforms on EGF25 were trisaccharides, while the majority of *O*-Glc glycoforms on EGF27 and EGF28 were monosaccharides. There were no *O*-Glc glycans detected on EGF9. Notably, EGF10 appeared to be modified with *O*-Glc glycans extended not only with two xylose residues but also with a hexose and a sialic acid.

Similarly, 70% or more of 12 out of 17 EGF repeats of NOTCH2 turned out to be modified with xylose-extended *O*-Glc glycans (Figure 3, Figure S2, Table S2). EGF4, EGF10, and EGF36 were modified with xylose-extended *O*-Glc glycans less efficiently than the 12 EGF repeats above. The majority of the xylosylated *O*-Glc glycans on EGF4 were disaccharides, whereas those on EGF10 and EGF36 were monosaccharides, suggesting that EGF4 of NOTCH2 may be a poor substrate for XXYLT1. Like EGF9 of NOTCH1, there was no *O-*Glc glycans detected on EGF23 of NOTCH2.

In order to gain an insight into the responsible xylosyltransferases for the addition of xylose residues on the *O*-Glc glycans in NOTCH1 and NOTCH2, we sought to delete both *GXYLT1* and *GXYLT2*, or *XXYLT1* in HEK293T cells genetically. Initially, we confirmed the mRNA expression of these genes in the cell line (Figure S3A). Thus, it was possible that both *GXYLT1* and *GXYLT2* contribute to the addition of the first xylose residues redundantly. A successful genome editing at the expected regions in these genes was confirmed by a genomic DNA sequencing (Figure S3B). The mass spectrometric analyses demonstrated that NOTCH1 or NOTCH2 produced in *GXYLT1* and *GXYLT2* double knockout (KO) cells did not contain any xylosylated *O*-Glc glycans, and that NOTCH1 or NOTCH2 produced in *XXYLT1* KO cells did not contain *O*-Glc trisaccharides with two xylose residues (Figure 2 and Figure 3). There was no substantial effect on the other types of *O*-glycosylation, such as *O*-fucose and *O*-GlcNAc (Figures S4–S6, Tables S3 and S4). Co-transfection of the NOTCH1 expression vectors with the GXYLT1 and GXYLT2 expression vectors in the *GXYLT1* and *GXYLT2* double KO cells or with the XXYLT1 expression vector in the *XXYLT1* KO cells rescued the xylosylation of *O*-Glc glycans on NOTCH1 (Figure S7). These results indicate that GXYLT1 and/or GXYLT2 are responsible for the addition of the first xylose, while XXYLT1 is responsible for the addition of the second xylose on *O*-Glc glycans of the NOTCH1 and NOTCH2 EGF repeats in HEK293T cells.

### 3.2. Xylosyl Extension of O-Glc Glycans Is Dispensable for Endogenous NOTCH1 and NOTCH2 Expression on the Cell Surface, But Required for the Trafficking of NOTCH1 and NOTCH2 Expressed at High Levels

**Figure 4:**
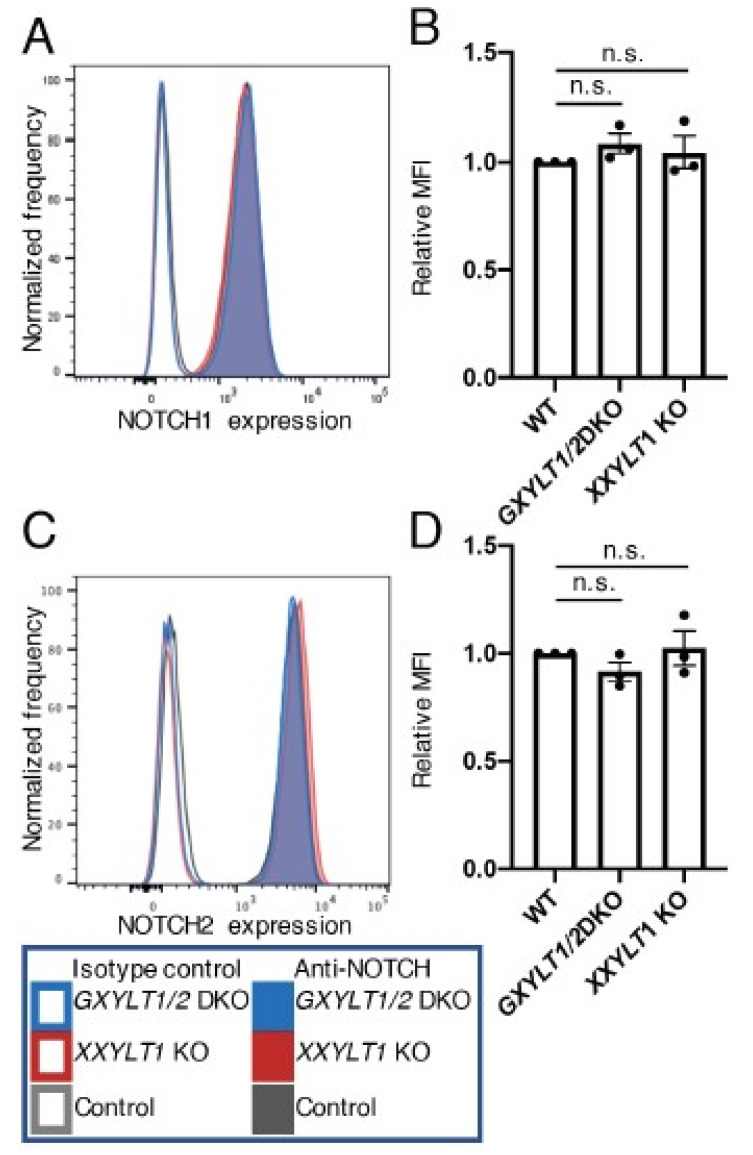
XYLTs are not required for the cell surface expression of endogenous NOTCH1 and NOTCH2 in HEK293T cells. (**A**) Histograms of endogenous NOTCH1 expression in wild type and *XYLTs*’ KO clones of HEK293T cells analyzed by flow cytometry. (**B**) Mean fluorescence intensity from (**A**). Plots are from three independent experiments (*n* = 3). Error bar denotes the standard error of the mean (SEM). Bar graphs show the average ± SEM. (**C**) Histograms of endogenous NOTCH2 expression in wild type and *XYLTs*’ KO clones of HEK293T cells analyzed by flow cytometry. (**D**) Mean fluorescence intensity from (**C**). Plots are from three independent experiments (*n* = 3). Bar graphs show the average ± SEM. The cell numbers on the vertical axis of the graphs for (**A**,**C**) are normalized with the mode value. n.s., not significant (*p* > 0.05).

The genetic deletion of POGLUT1 reduced the expression of NOTCH1 on the surface of HEK293T cells by approximately 50% [17]. Since the loss of POGLUT1 was expected to have wiped out the *O*-Glc modifications on NOTCH1, it remained possible that xylosyl extension plays a key role in the expression of endogenous NOTCH1 on cell surfaces. We investigated the cell surface expression of endogenous NOTCH1 or NOTCH2 in the wild type control, *GXYLT1* and *GXYLT2* double KO cells, and the *XXYLT1* KO HEK293T cells by flow cytometry using antibodies specific against each receptor. No significant differences in the levels of NOTCH1 or NOTCH2 on the cell surface in the wild type control or KO cells were observed. These results strongly suggest that the xylosyl extension of *O*-Glc glycans is not necessary for the trafficking of NOTCH1 and NOTCH2 in HEK293T cells when expressed at an endogenous level (Figure 4).

**Figure 5:**
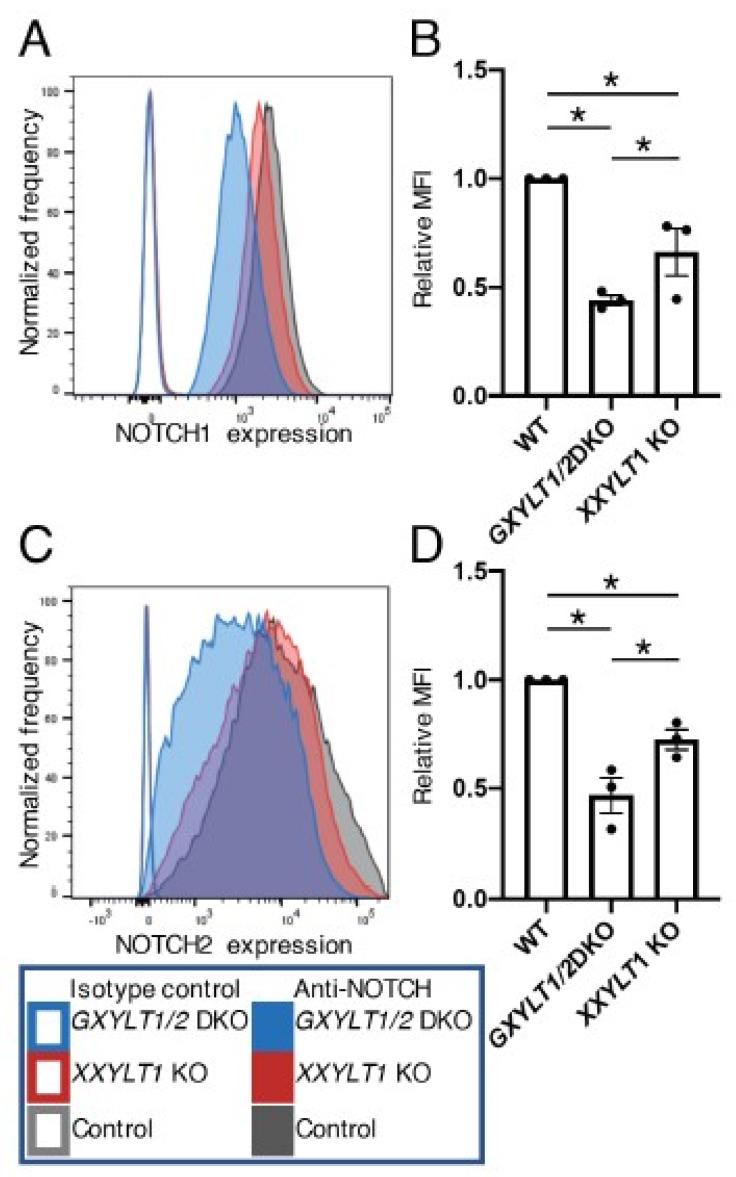
Xylosyl extension of *O*-Glc glycans enhances the cell surface expression of NOTCH1 or NOTCH2 overexpressed in HEK293T cells. (**A**) The cell surface expression of transfected full length NOTCH1 in the wild type and XYLTs’ KO clones of HEK293T cells analyzed by flow cytometry. Co-transfected GFP-positive cells were gated. (**B**) Mean fluorescence intensity from (**A**). Plots are from four independent experiments (*n* = 4). Bar graphs show the average ± SEM. (**C**) Cell surface expression of transfected full length NOTCH2 in the wild type and *XYLTs*’ KO clones of HEK293T cells analyzed by flow cytometry. Co-transfected GFP-positive cells were gated. (**D**) Mean fluorescence intensity from (**C**). Plots are from three independent experiments (*n* = 3). Bar graphs show the average ± SEM. The cell numbers on the vertical axis of the graphs in (**A**,**C**) are normalized with the mode value. *, *p* < 0.05.

Then, we overexpressed *N*-terminally FLAG-tagged full length NOTCH1 and *C*-terminally FLAG-tagged full length NOTCH2 with GFP in the cells and examined the expression of NOTCH1 and NOTCH2 by flow cytometry using an anti-FLAG antibody and anti-mouse NOTCH2 antibody, respectively. For a fair comparison, the transfection efficiency was normalized by gating GFP-positive cells when we analyzed the NOTCH expression levels in all cells. The expression of NOTCH1 and NOTCH2 on the surface of the *GXYLT1* and *GXYLT2* double KO cells and *XXYLT1* KO cells was significantly lower than that in the wild type control cells (Figure 5).

**Figure 6:**
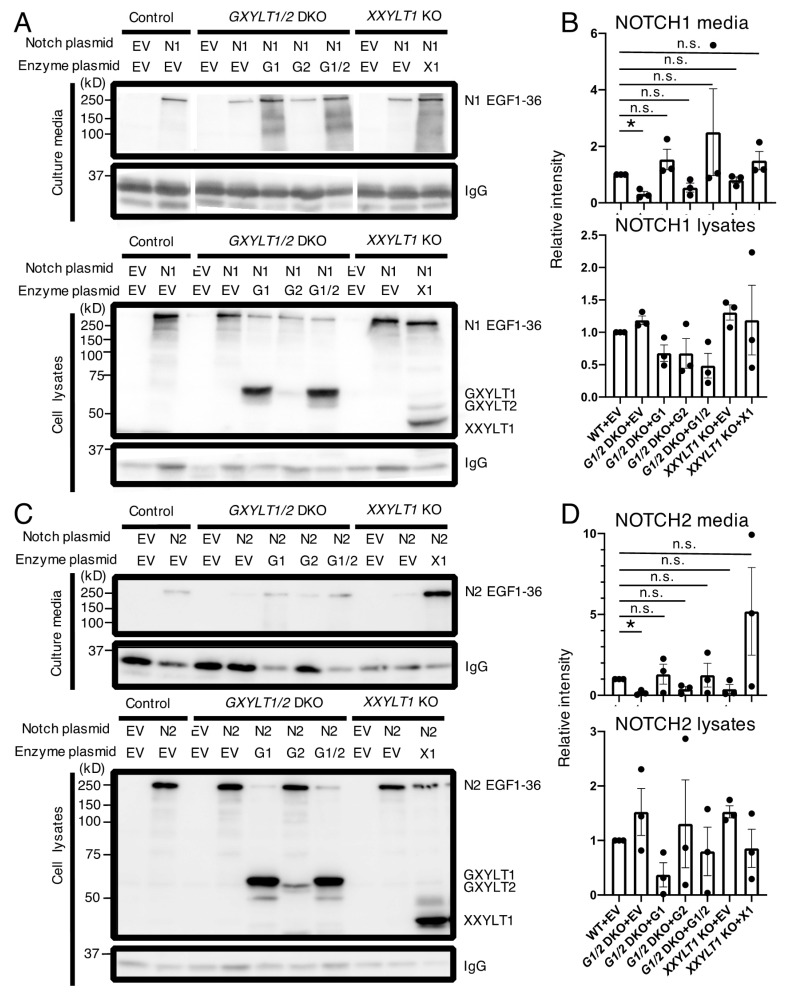
Xylosyl extension of *O*-Glc glycans enhances the secretion of the ECDs of NOTCH1 and NOTCH2 overexpressed in HEK293T cells. (**A**) Secretion assay with the Myc-His_6_-tagged version of EGF1–36 of NOTCH1 (N1 EGF1–36) in the wild type control and *XYLTs*’ KO clones. The N1 EGF1–36 proteins in the culture media and the cell lysates were detected by Western blotting using an anti-Myc antibody. EV, empty vector; N1, NOTCH1; G1, GXYLT1; G2; GXYLT2, X1; XXYLT1. A representative image of three independent experiments is shown. (**B**) The relative intensities of the N1 EGF1–36 proteins are indicated. Plots are from three independent experiments (*n* = 3). Bar graphs show the average ± SEM. * *p* < 0.05; n.s., not significant. (**C**) Secretion assay with the Myc-His_6_-tagged version of EGF1–36 of NOTCH2 (N2 EGF1–36) in the wild type control and *XYLTs*’ KO clones. N2 EGF1–36 proteins in the culture media and cell lysates were detected by Western blotting using an anti-Myc antibody. EV, empty vector; N1, NOTCH1; G1, GXYLT1; G2; GXYLT2, X1; XXYLT1. A representative image of three independent experiments is shown. (**D**) The relative intensities of the N2 EGF1–36 proteins are indicated. Plots are from three independent experiments (*n* = 3). Bar graphs show the average ± SEM. The raw data are available in Figure S8. *, *p* < 0.05; n.s., not significant (*p* ≥ 0.05).

To further support that the requirement for the xylosyl extension of *O*-Glc glycans localizes to the EGF repeats in the ECD of NOTCH1 and NOTCH2, we performed cell-based secretion assays with *C*-terminally MycHis_6_-tagged versions of NOTCH1 and NOTCH2 constructs encoding EGF1–36 of each paralog, as well as the expression vector of IgG, which did not have EGF repeats, as a control. Previously, we performed the same assay system when we analyzed the *POGLUT1*-KO 293T cells with the same NOTCH1 EGF1–36 construct [17]. In the present study, the effect of deficiency of the xylosyltransferases, which modify *O*-Glc, was examined by including NOTCH2 as a new target in addition to NOTCH1. Compared with the wild type control cells, the levels of the NOTCH1 EGF1–36 proteins secreted to the culture media were lower in the *GXYLT1* and *GXYLT2* double KO cells (Figure 6A,B). Although not statistically significant, the secretion of the NOTCH1 ECD showed a slight decrease in the *XXYLT1* KO cells compared with the wild type control cells (Figure 6A,B). There was no statistically significant difference in the levels of the NOTCH1 EGF1–36 proteins in the cell lysates from each sample compared with those in the wild type control cells (Figure 6A,B). The Notch proteins in the KO cells might not be accumulated but instead degraded. Importantly, the secretion defects in the *GXYLT1* and *GXYLT2* double KO cells were rescued by the co-transfection of the GXYLT1 or GXYLT2 expression vector alone, or in combination (Figure 6A,B). Similar results were obtained for the NOTCH2 secretion (Figure 6C,D). These results indicated that the xylosyl extension of *O*-Glc glycans is required for the trafficking of NOTCH1 and NOTCH2 in HEK293T cells when expressed at high levels.

## 4. Discussion

In this study, we found that the majority of the EGF repeats of NOTCH1 and NOTCH2 are modified with *O*-Glc trisaccharides, while the remaining EGF repeats, such as EGF27 and EGF28 from NOTCH1 and EGF4, EGF10, EGF23, and EGF36 from NOTCH2, are not. The xylosyl extension of *O*-Glc glycans on NOTCH1 and NOTCH2 were found to be required for trafficking when the receptors were overexpressed in HEK293T cells.

Both NOTCH1 and NOTCH2 have 17 EGF repeats with the *O*-Glc consensus sequence (C1-X-S-X-P/A-C2). Previous studies found that most of the EGF repeats on mouse NOTCH1 could be modified with *O*-Glc trisaccharides [26]; however, they did not address the stoichiometries on the EGF repeats on NOTCH1. Our data indicated that an *O*-Glc modification on NOTCH1 occurs at high stoichiometries on most of the EGF repeats with the *O*-Glc consensus sequence, except for EGF9, EGF25, EGF27, and EGF28. Furthermore, we found that the xylosyl extension of *O*-Glc glycans on NOTCH1 is site-specific. The EGF9 of NOTCH1 has an irregular consensus sequence, with an alanine residue just before the second cysteine residue. A previous enzymatic analysis of POGLUT1 showed that a substitution from a proline residue to an alanine residue resulted in an EGF repeat with a worse substrate [32]. Recent X-ray structural studies on *Drosophila* rumi (POGLUT1) and human POGLUT1 co-crystalized with substrates support this finding [33,34]. The POGLUT1 structures from the two different species were well-conserved. The proline residue within the *O*-Glc consensus sequence is critical not only for the hydrophobic interaction with POGLUT1 but also for the formation of the U-like configuration of an EGF repeat, which results in the *O*-Glc modification site being inserted deeply into the POGLUT1 active site. EGF27 was shown to be a poor substrate for POGLUT1, partly due to the arginine residue at the +1 position within the *O*-Glc consensus sequence. These findings suggest that the stoichiometry of the *O*-Glc modification at each EGF repeat may highly depend on the amino acid sequence of the individual EGF repeats.

What determines the extent of xylosyl extension on individual EGF repeats of Notch receptors? Enzymatic assays with an *O*-glucosylated synthetic peptide analog were used to originally detect GXYLT activity [24]. Folded EGF repeats modified with *O*-Glc were shown to be better substrates for GXYLT1, GXYLT2, and XXYLT1 than unfolded ones [32]. Previous studies have suggested that the xylosyltransferases recognize the aglycone structure of the acceptor substrate. In *Drosophila* and S2 cells, where a sole GXYLT, Shams is expressed, the levels of the xylosyl extension of *O*-Glc glycans are higher in the middle region of the *Drosophila* Notch, and only a limited number of EGF repeats in that region are modified with *O*-Glc trisaccharides [35]. The comparison of the amino acid sequence of the EGF repeats failed to provide a clear rule for the regulation of the xylosyl extension on individual EGF repeats in the *Drosophila* Notch [35]. GXYLT1 and GXYLT2 may not only recognize a single EGF repeat with an *O*-Glc monosaccharide but also a greater region in a cellular context. However, further systematic analysis is necessary.

We identified previously unreported *O*-linked glycans at specific EGF repeats including EGF10 of NOTCH1 derived from HEK293T cells. Namely, our mass spectral data on *O*-Glc glycans in NOTCH1 and NOTCH2 indicated that the novel linear, hexosylated structures contained a hexose and a sialic acid in the most extended form. This notion raises many interesting and important questions. For example, what kind of hexose residues exist in these glycoforms and which glycosyltransferases are involved in the biosynthesis of the new structures? Even in the absence of *GXYLT1* and *GXYLT2*, the novel glycans are formed in the limited numbers of EGF repeats such as EGF12 in NOTCH1, and EGF12 and EGF33 in NOTCH2 (Figure 2 and Figure 3), suggesting the acceptor specificity of the responsible hexosyl-transferase(s). *O*-Fuc and *O*-GlcNAc glycans on Notch EGF repeats are known to contain extended structures with a galactose and a sialic acid [19,27,29,35]. Several lines of evidence indicated that the addition of galactose residues on Fringe-modified *O*-Fuc glycans modulates Jagged1-mediated Notch signaling in specific contexts [36,37,38]. A previous study using the Lec2 Chinese hamster ovary cell mutant defective in addition to sialic acids suggested that the sialic acid is not required for Fringe to modulate Notch signaling [36]. Nonetheless, given that the novel hexyosyl extension of *O*-Glc glycans in the EGF repeats exists in the Notch ligand-binding region [39,40,41] and that endogenous lectins such as galectins are implicated in the Notch signaling pathway [42], it will be important to investigate the structure and function of the novel glycans in Notch receptors.

**Figure 7:**
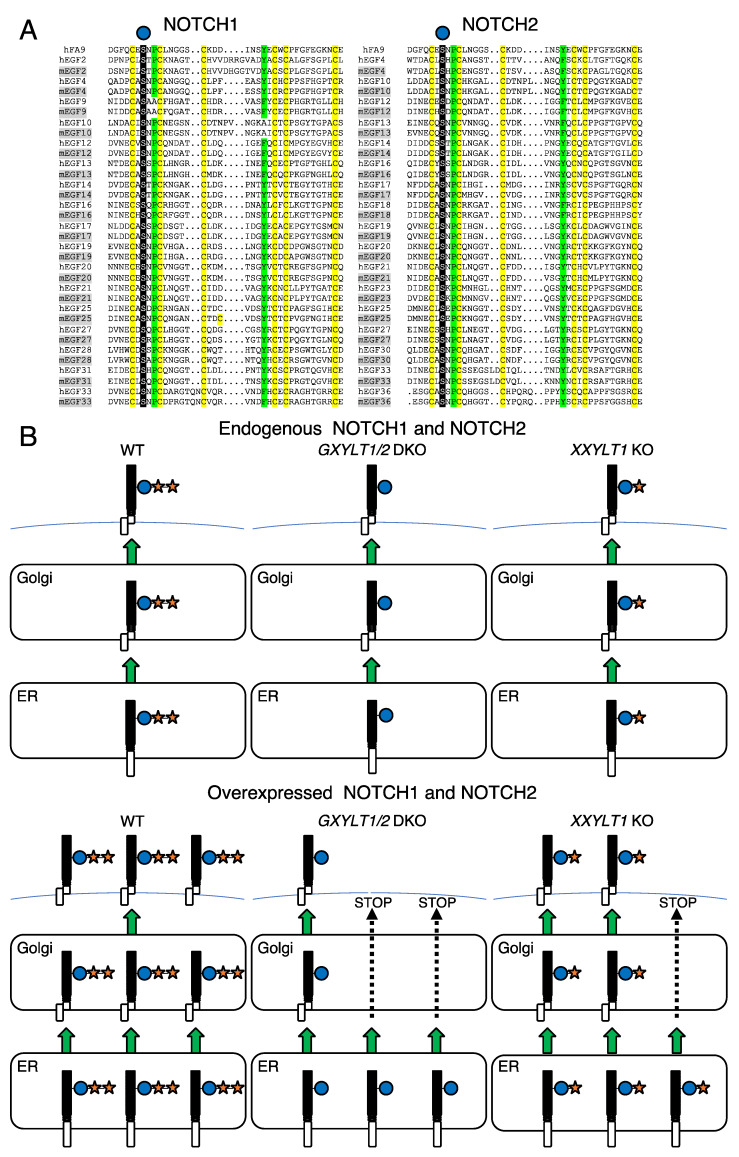
The potential role of xylosyl extension of *O*-Glc glycans in the quality control of NOTCH1 and NOTCH2. (**A**) Sequence alignment of all 17 EGF repeats of human and mouse NOTCH1 (left column) and NOTCH2 (right column) harboring the *O*-Glc consensus sequence with the EGF repeat 1 from human factor IX (hFA9). The *O*-Glc modification site within the consensus sequence is highlighted in black and indicated with a blue circle. Six conserved cysteine residues are highlighted in yellow. An *O*-Glc trisaccharide is shown to interact intramolecularly with the hydrophobic region formed by Proline 55 and Tyrosine 69 in the hFA9 EGF repeat (17). The corresponding positions are highlighted in green. (**B**) Schematic presentation of the effects of the loss of *XYLTs* on the cell surface expression of NOTCH1 and NOTCH2. The loss of *XYLTs* in HEK293T cells did not cause any change in the cell surface expression of endogenous NOTCH1 and NOTCH2 (top). When NOTCH1 and NOTCH2 are overexpressed, the loss of both *GXYLT1* and *GXYLT2* significantly decreased their cell surface expression, while the loss of *XXYLT1* showed a milder, but substantial effect (bottom).

In a previous study, we showed that POGLUT1 and POFUT1 were required for the normal trafficking of endogenous NOTCH1 in HEK293T cells [17]. Both POGLUT1 and POFUT1 are localized in the ER and modify the EGF repeats folded properly with three disulfide bonds in a specific pattern [20,22,43]. The addition of an *O*-Glc monosaccharide and an *O*-Fuc monosaccharide stabilizes an EGF repeat via intramolecular interactions in an additive manner [17]. Based on these findings, we proposed that *O*-Glc glycans are involved in the quality control of EGF-containing proteins (e.g., Notch) by stabilizing the EGF repeats, as is the case for the *O*-fucosylation of thrombospondin type-I repeat (TSR)-containing proteins (e.g., ADAMTS proteases) [16,44,45,46]. The same study revealed that the addition of the second, but not the first, xylose residue stabilizes a single EGF repeat [17]. The sequence alignment of the EGF repeats with the *O*-Glc consensus sequence from NOTCH2 demonstrated the high conservation of amino acids that potentially form intramolecular interactions with *O*-Glc trisaccharides, as in the crystallized structure of the human factor IX EGF repeat modified with an *O*-Glc trisaccharide (Figure 7A) [17]. Therefore, it would be a reasonable assumption that *O*-Glc glycans stabilize the EGF repeats from NOTCH2 as well as NOTCH1. It is worth noting that the presence of the *O*-Glc consensus and the amino acid sequence of the EGF repeats are well-conserved in humans and mice in each Notch paralog (Figure 7A).

Both POGLUT1 and XXYLT1 are localized in the ER [20,22,25], which suggests that the biosynthesis of the xylosyl extension of *O*-Glc glycans is likely to be initiated and finished in the ER. Thus, to determine the potential role of the xylosyl extension of *O*-Glc glycans in the quality control of proteins, we investigated the cell surface expression of full length NOTCH1 and NOTCH2 when the xylosyltransferase genes were knocked out in HEK293T cells. Upon the loss of the xylosyl extension of *O*-Glc glycans in the HEK293T cells, we did not observe any defects in the trafficking of endogenous NOTCH1 or NOTCH2, whereas we did observe a defect in the trafficking of overexpressed NOTCH1 and NOTCH2. These findings may suggest that the stabilizing effect of the xylosyl extension on multiple EGF repeats in the ECDs of NOTCH1 and NOTCH2 promotes the efficient trafficking of NOTCH1 and NOTCH2 to the cell surface (Figure 7B). In our overexpression experiments of NOTCH1 and NOTCH2, we co-transfected GFP or IgG in the flow cytometric analysis or the secretion assay, respectively. Even with controls, we cannot rule out the possibility that changes caused by the overexpression of a Notch could have affected the outcome. Such things may have something to do with glycosylation. For example, the details of the molecular species and expression levels involved in the formation and folding of disulfide bonds in the EGF repeats have not been clarified, and these factors need to be clarified in the future. Another potential caveat in these experiments is, although this is not our favorable model, that there may be unidentified molecules harboring the EGF repeats with the *O*-Glc consensus sequence that regulate the trafficking of Notch receptors. Furthermore, it is not clear whether the novel hexosyl extension found at specific EGF repeats including EGF10 in NOTCH1 can stabilize the EGF repeats yet. It should be noted that an alanine mutation of the predicted *O*-Glc site at EGF10 in NOTCH1 did not cause any defect in the cell surface expression of NOTCH1 [25].

The function of Notch receptors, which play a central role in Notch signaling, is regulated at various levels in the cell, and glycosylation may be one of them [2,3,6,7,8,9,10]. The expression levels of Notch receptors vary with each tissue and cell type [47], and it is also known that Notch1 expression levels oscillate during the formation of somites, termed somitogenesis, during development [48]. Fringe, which modifies *O*-fucose, is known to be involved in the regulation of this process [49,50]. It is also known that Notch signaling is involved in the maintenance of stem cells in many adult tissues in mammals, and *O*-Glc glycosylation is likely to be involved in these events. Indeed, an abnormal somite formation has been observed in *Poglut1* knockout mice [21], and we have recently shown that a reduced enzymatic activity of POGLUT1 in satellite cells, the stem cells of muscles, leads to a reduced Notch signaling, resulting in the aberrant maintenance and differentiation of satellite cells, which induces muscular dystrophy [51,52]. Multiple NOTCH receptors, particularly NOTCH2, are important for satellite cell function in muscles, and their expression levels have been reported to decrease with differentiation [53,54]. In this study, we only showed that the xylosyl extension of *O*-Glc glycans may be involved in the regulation of Notch receptor trafficking using an overexpression system with room for improvement, and it should be further investigated how a xylosyl extension is involved in the above-mentioned, and possibly other, biological processes in vivo. Taken together, our findings suggest the importance of protein folding and *O*-glucosylation on multiple cysteine-rich EGF repeats of Notch receptors and provide insights into the pathological mechanisms in *POGLUT1*-related human diseases [51,52,55,56,57].

## Supplementary Materials

The following are available online at https://www.mdpi.com/2073-4409/9/5/1220/s1, Figures S1 and S2: MS/MS spectra of (glyco)peptides from NOTCH1 and NOTCH2, Figure S3: *GXYLT1*, *GXYLT2*, and *XXYLT1* are expressed in HEK293T cells, Figure S4: Knockout of the xylosyltransferases does not affect the attachment and elongation of *O*-fucose and *O*-GlcNAc glycans, Figure S5: MS/MS spectra of (glyco)peptides with the predicted O-fucose site from NOTCH1, Figure S6: MS/MS spectra of (glyco)peptides with the predicted *O*-GlcNAc site from NOTCH1, Figure S7: Co-transfection of XYLTs with NOTCH1 rescues xylosylation on NOTCH1, Figure S8: The raw data for Figure 6 “Xylosyl-extension of *O*-Glc glycans enhances the secretion of the ECDs of NOTCH1 and NOTCH2 overexpressed in HEK293T cells”, Table S1: Summary of mass spectral semi-quantification of NOTCH1 *O*-Glc glycans, Table S2: Summary of mass spectral semi-quantification of NOTCH2 *O*-Glc glycans, Table S3: Summary of mass spectral semi-quantification of NOTCH1 *O*-fucose glycans, Table S4: Summary of mass spectral semi-quantification of NOTCH1 *O*-GlcNAc glycans.
