# Supplementary material for: Xylosyl Extension of O-Glucose Glycans on the Extracellular Domain of NOTCH1 and NOTCH2 Regulates Notch Cell Surface Trafficking
Source: Cells. 2020 May 14;9(5):1220. doi: 10.3390/cells9051220 (PMC7291291; doi:10.3390/cells9051220)
Supplement: Supplementary file 1 [file cells-09-01220-s001.pdf]

Supplementary Materials

Xylosyl-extension of *O*-glucose glycans on the extracellular domain of NOTCH1 and NOTCH2 regulates Notch cell surface trafficking

Yusuke Urata [1,2], Wataru Saiki [1], Yohei Tsukamoto [1], Hiroaki Sago [1], Hideharu Hibi [2], Tetsuya Okajima [1,*], and Hideyuki Takeuchi [1,*]

Figures

Figure S1-1

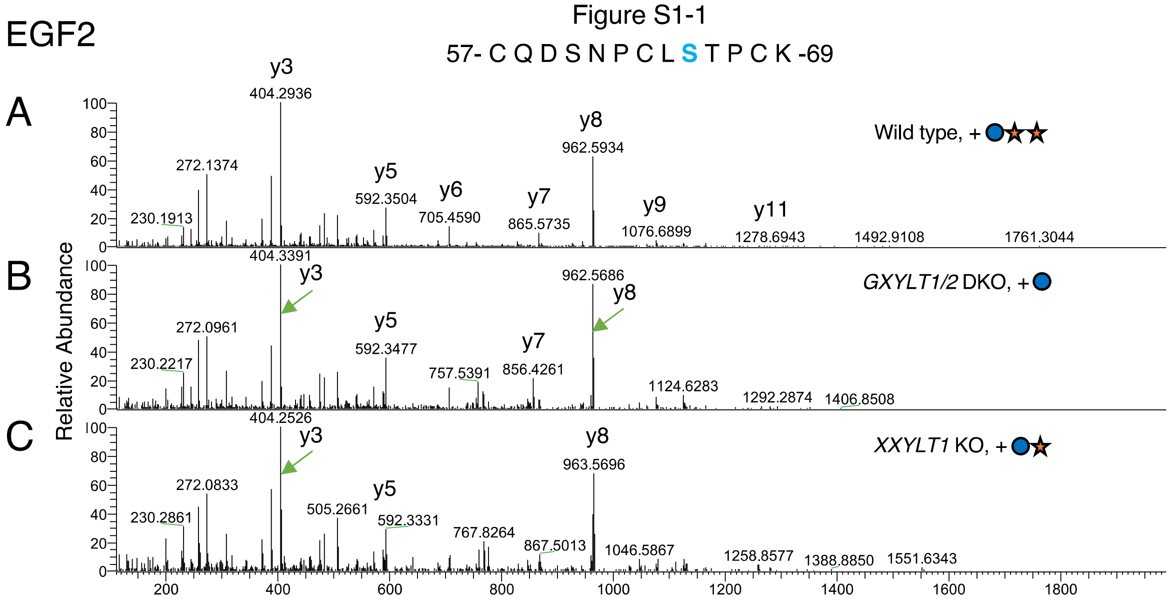

Figure S1-2

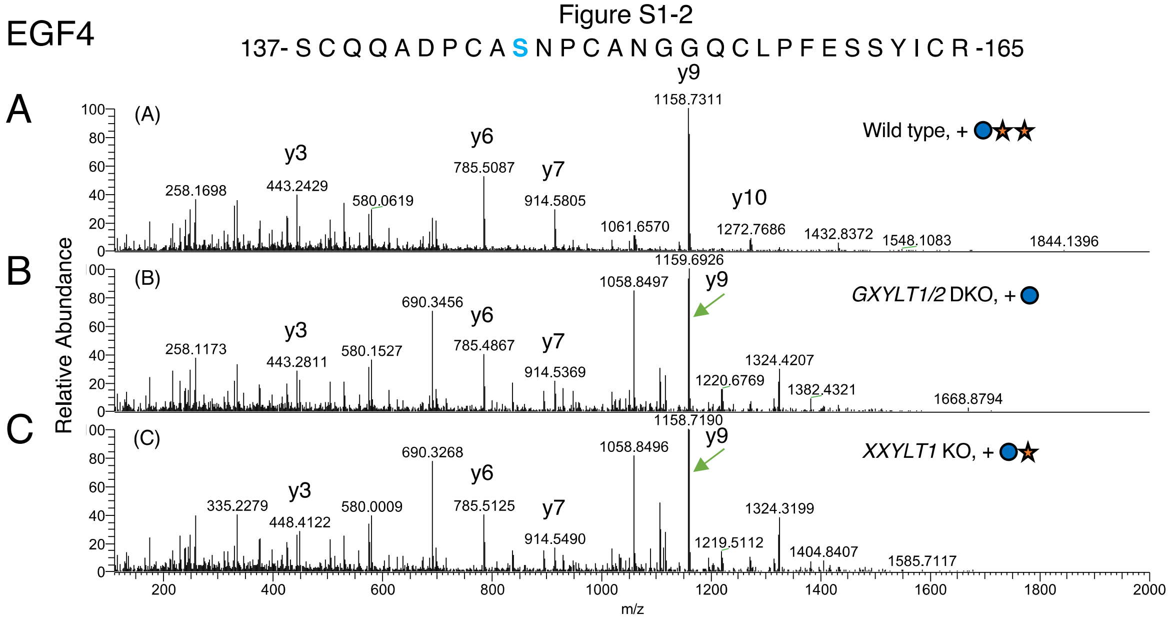

Figure S1-3

EGF9

328- T G E D C S E N I D D C A S A A C F -345

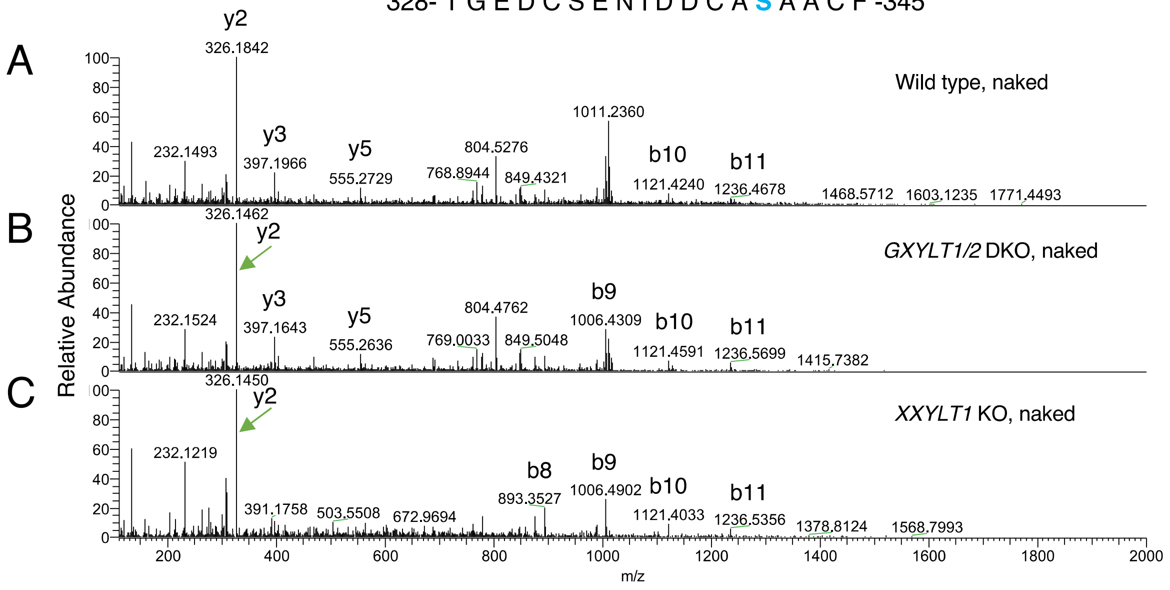

Figure S1-4

EGF10

366- T G L L C H L N D A C I S N P C N E G S N C D T N P V N G K -395

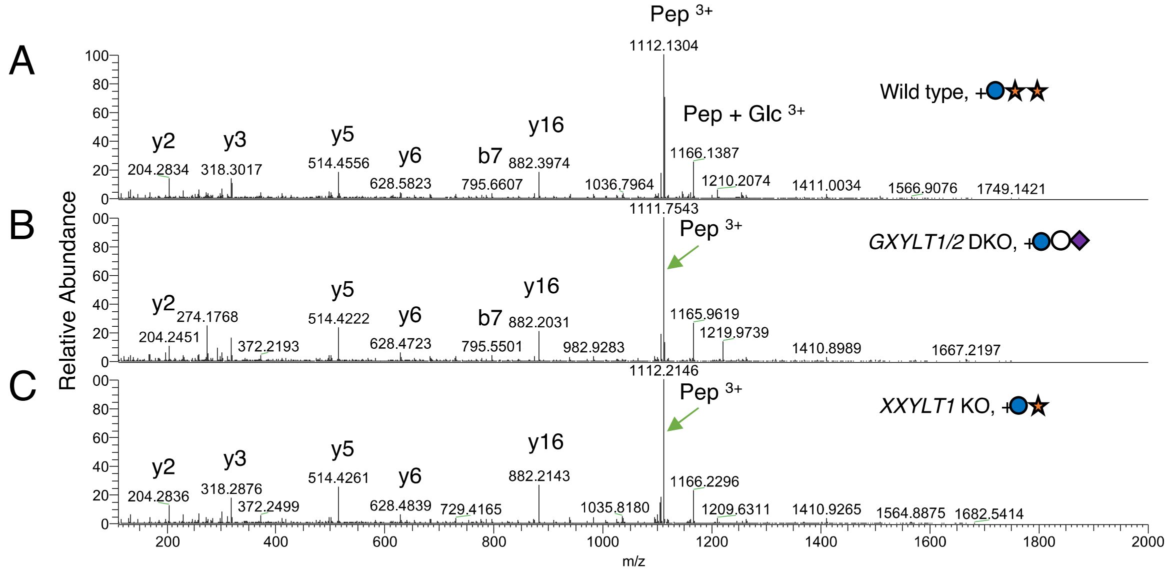

EGF12

Figure S1-5

445- T G P R C E I D V N E C I **S** N P C Q N D A **T** C L D Q I G E F -474

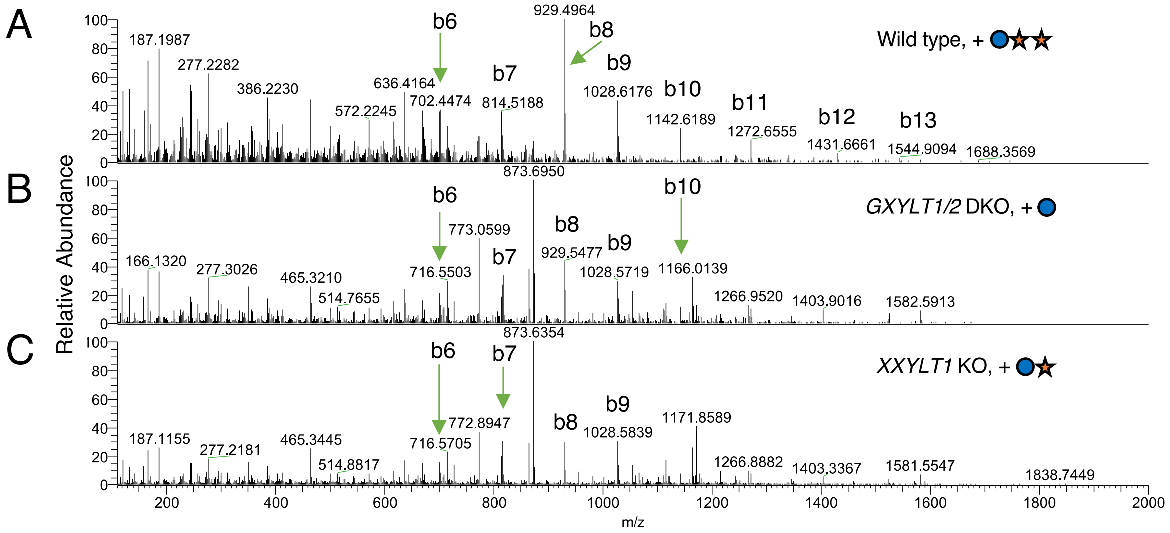

Figure S1-6

EGF13

487- C E I N T D E C A **S** S P C L H N G H C M D K N E F-512

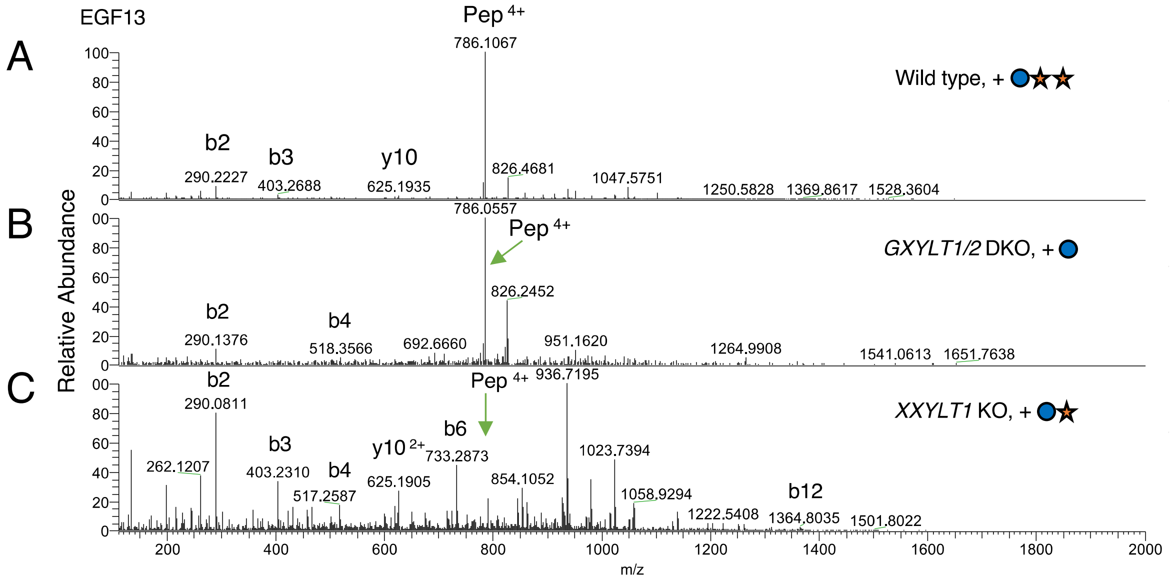

EGF14

Figure S1-7

519- G F N G H L C Q Y D V D **S** T P C K -538

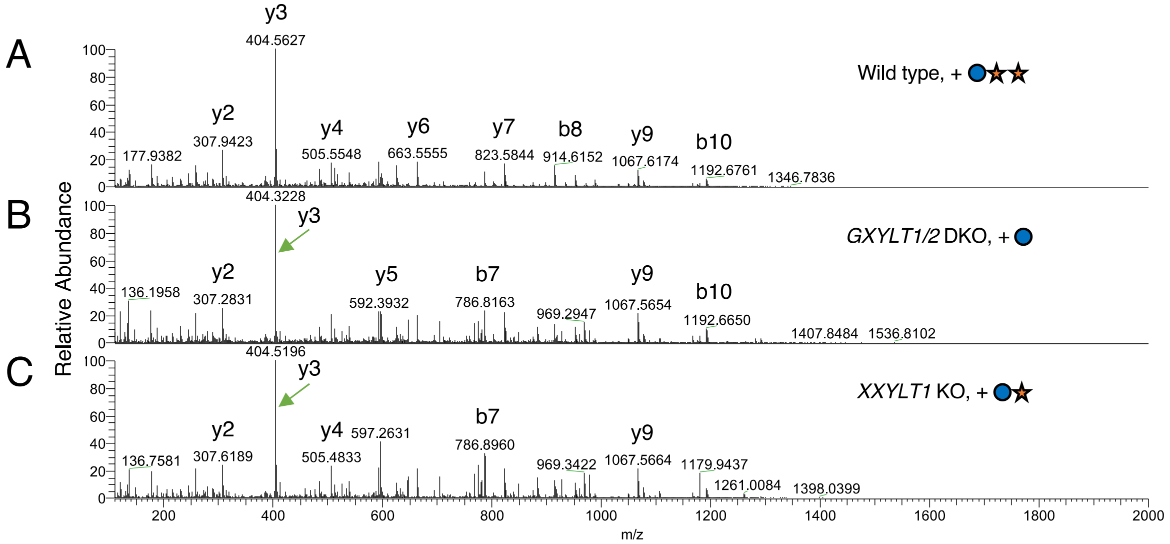

EGF16

Figure S1-8

582- D G V A T F T C L C Q P G Y T G H H C E T N I N E C H **S** Q P C R -613

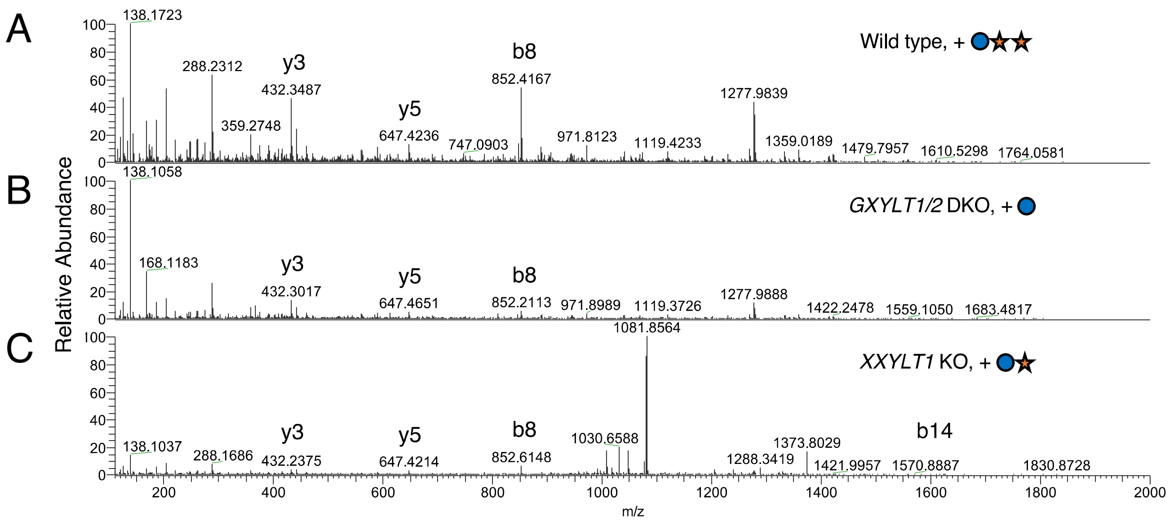

Figure S1-9

EGF17

632- G T T G P N C E I N L D D C A S N P C D S G T C L D K -658

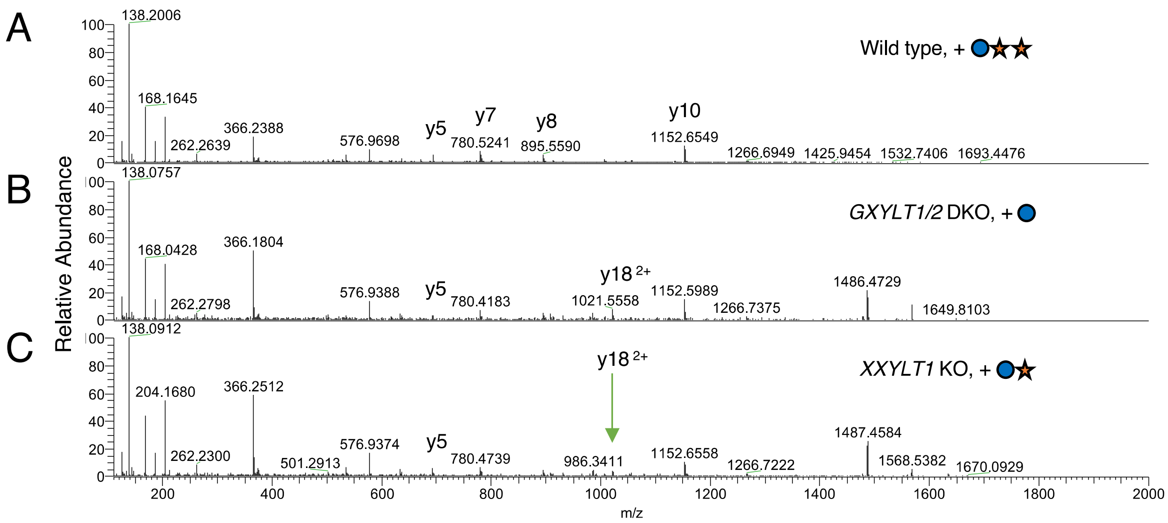

Figure S1-10

EGF19

709- T Y E A S L S E V N E C N S N P C I H G A C R -731

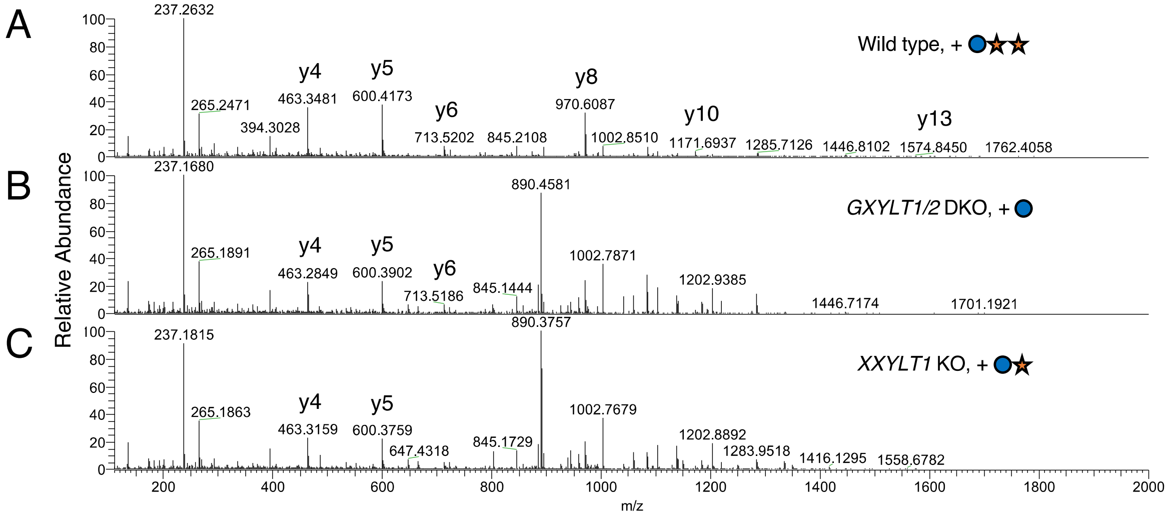

Figure S1-11

EGF20

739- C D C A P G W S G T N C D I N N N E C E S N P C V N G G T C K -769

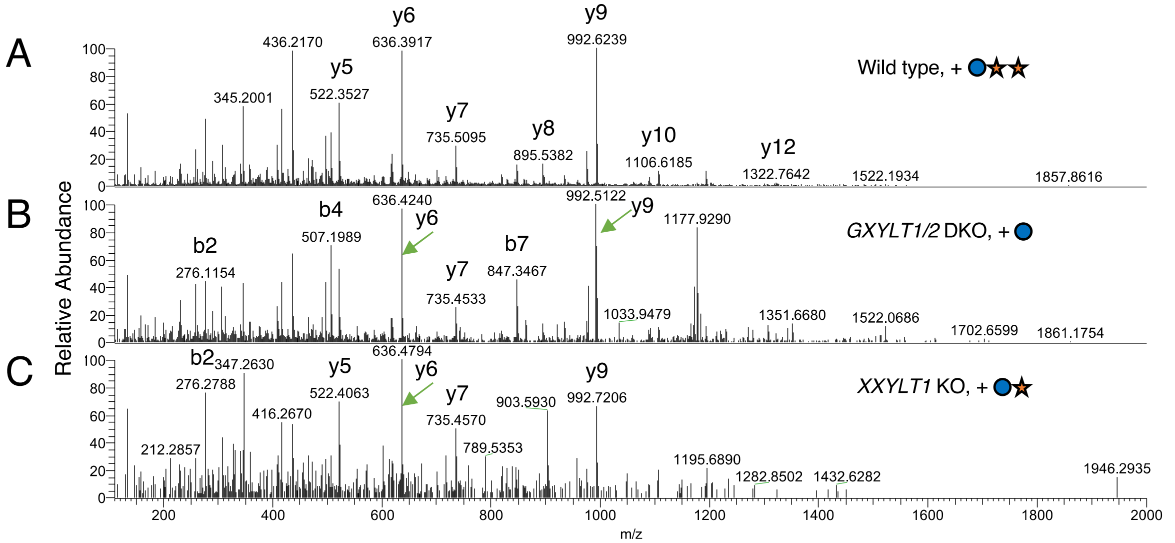

Figure S1-12

EGF21

781- E G F S G P N C Q T N I N E C A S N P C L N Q G T C I D D V A G Y K -814

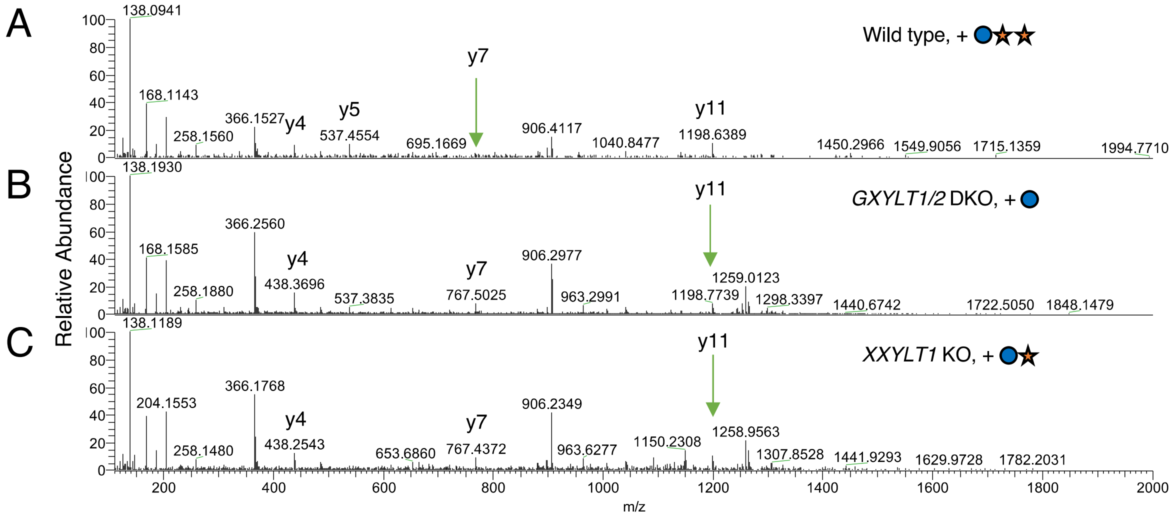

EGF25

Figure S1-13

942- C E E D I N E C A S N P C Q N G A N C T D C V D S Y -967

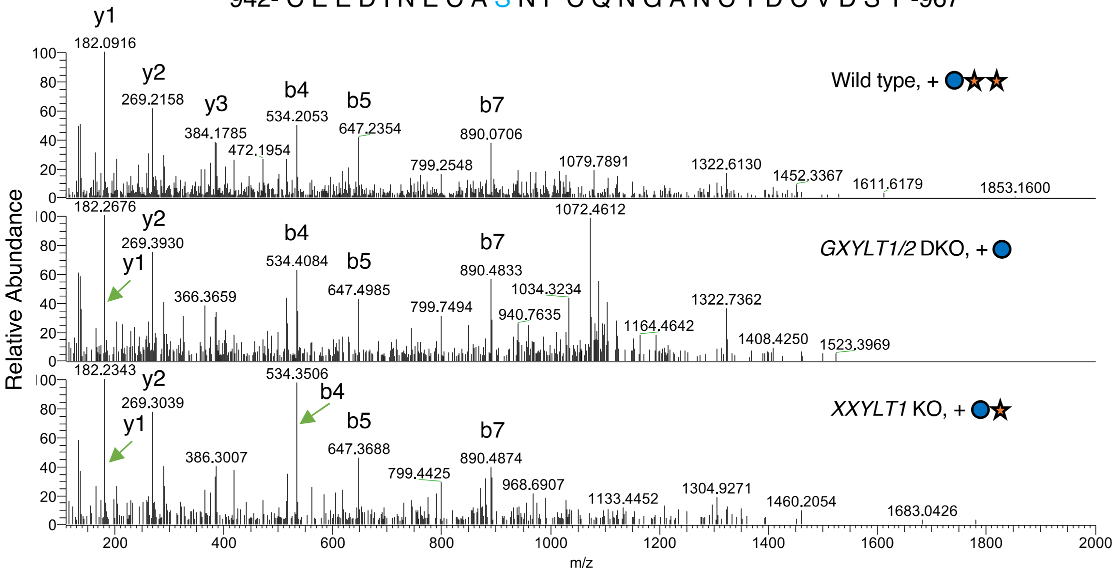

EGF27

Figure S1-14

1021- D V N E C D S R P C L H G G T C Q D S Y -1040

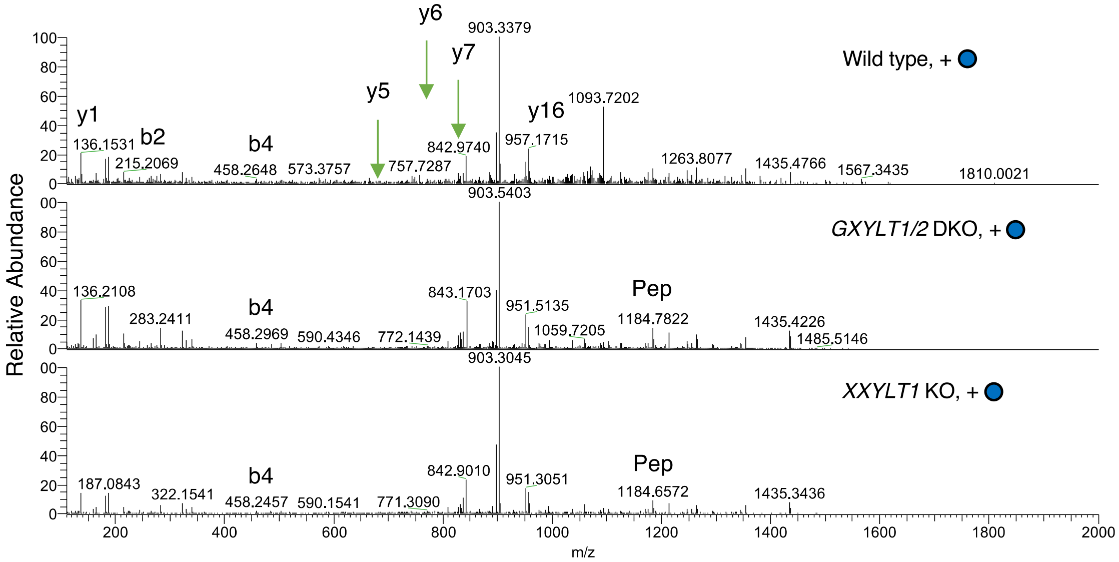

EGF28

Figure S1-15

1062- W C D S A P C K -1069

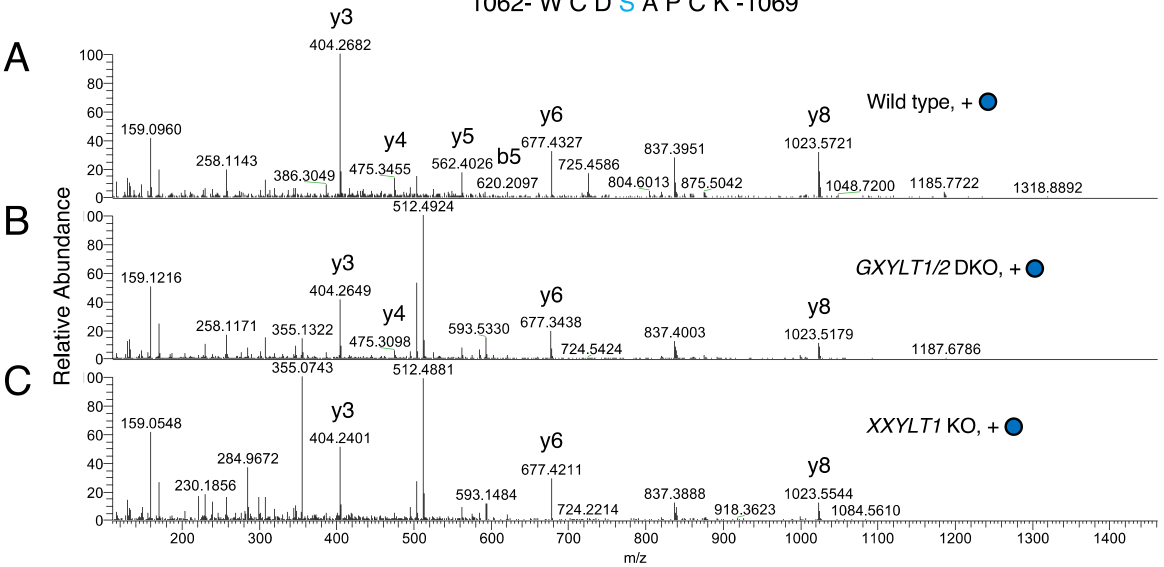

EGF31

Figure S1-16

1176- H G S N C S E E I N E C L S Q P C Q N G G T C I D L T N S Y-1205

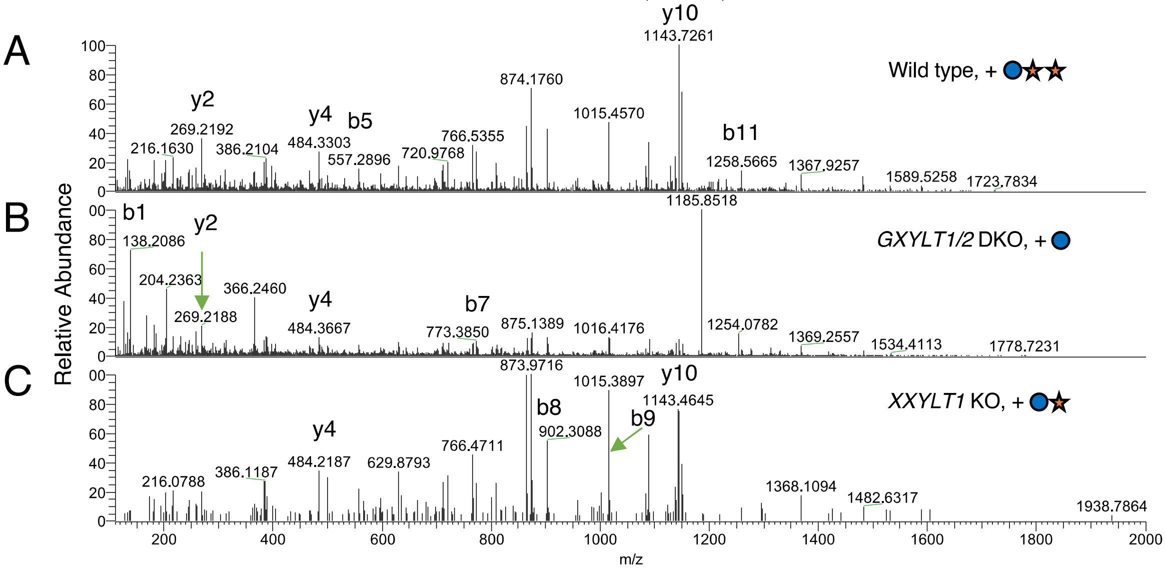

EGF33

Figure S2117

1264- C E G D V N E C L S N P C D P R -1279

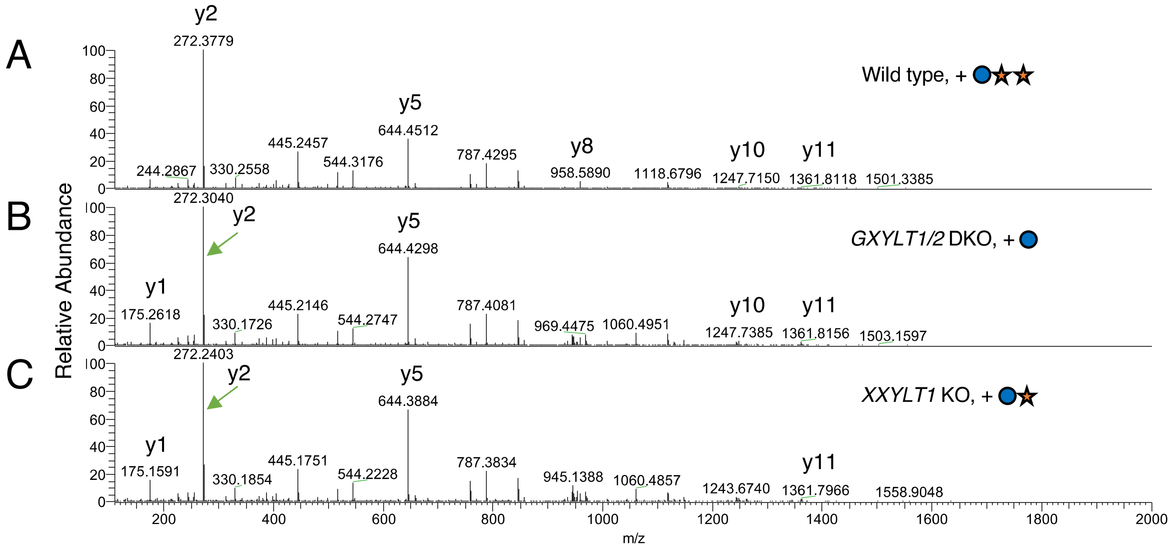

Figure S1. MS/MS spectra of (glyco)peptides from NOTCH1. MS/MS spectra of (glyco)peptide from NOTCH1. Samples were generated in wild type control HEK293T cells (A), *GXYLT1/2* DKO cells (B), *XXYLT1* KO cells (C) transfected with the plasmids encoding mouse NOTCH1 ECDs as described in Materials and Methods. The data in Figure S1-1 through S1-9 are derived from the analysis of mouse NOTCH1 EGF1-18. The data in Figure S1-10 through S1-12, S1-15 through S1-17 are derived from the analysis of mouse NOTCH1 EGF19-36. The data in Figure S1-13, and S1-14 are derived from the analysis of mouse NOTCH1 EGF24-28. MS/MS spectra confirmed the identity of (glyco)peptides based on the presence of peptide specific b- and y- ions and neutral loss of predicted glycans. Amino acid sequences are shown with the identified fragment ions at the upper right corner. For each EGF repeat from NOTCH1, MS/MS spectra of glycopeptides modified with the major glycoform derived from each cell clone are shown. The sequence of peptides, the predicted and measured m/z, and the charge state are summarized in Table S1.

EGF4

Figure S2-1

141- Q C Q W T D A C L S H P C E N G S T C T S V A S Q F S C K -169

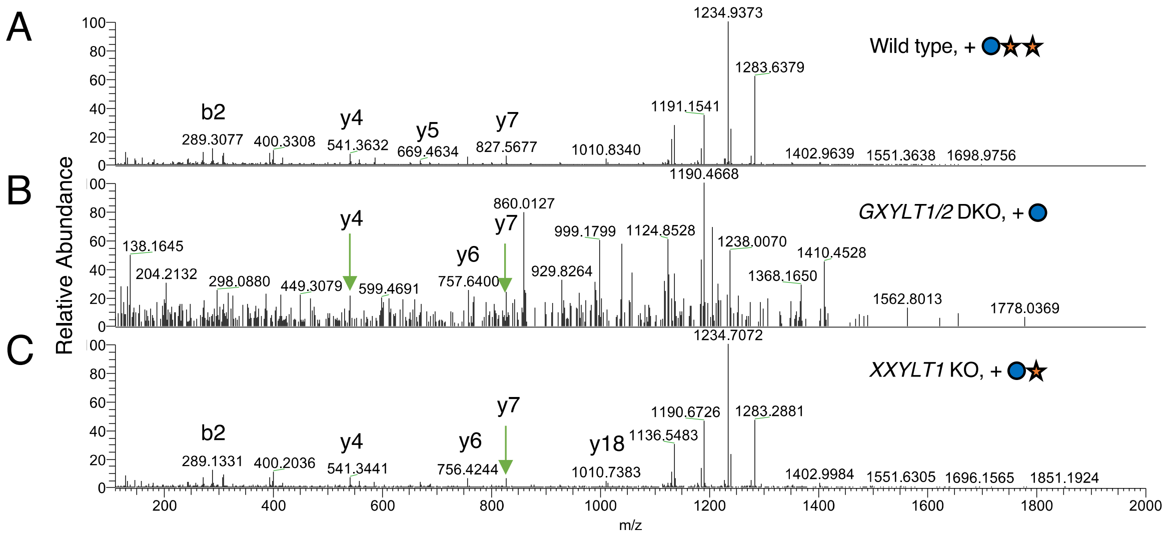

EGF10

Figure S2-2

369- A G L L C H L D D A C I S N P C H K -386

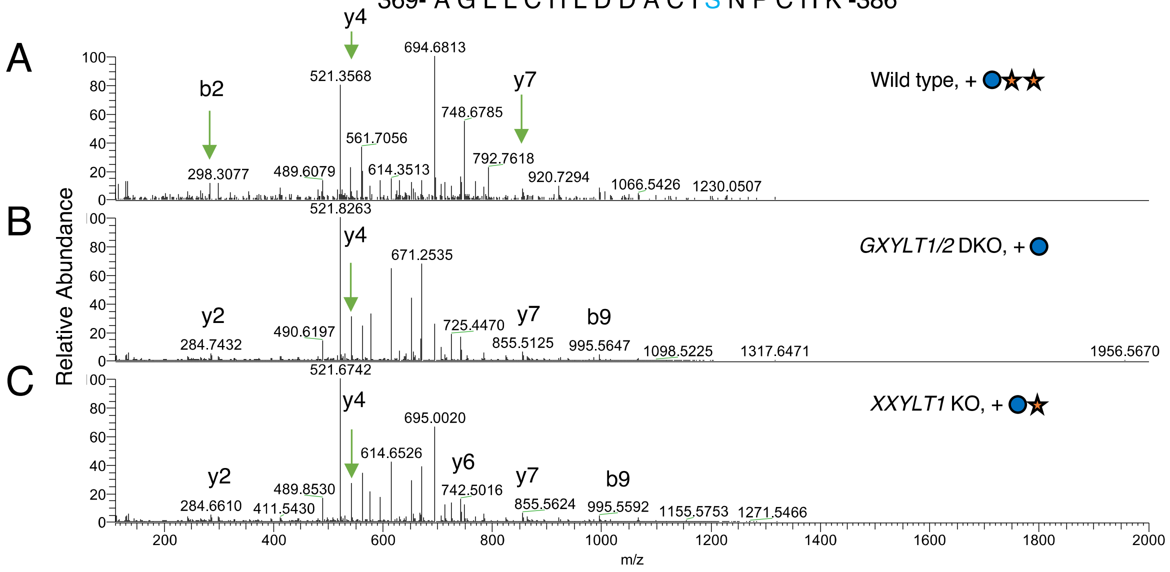

EGF12

Figure S2-3

453- C E M D I N E C H S D P C Q N D A T C L D K -474

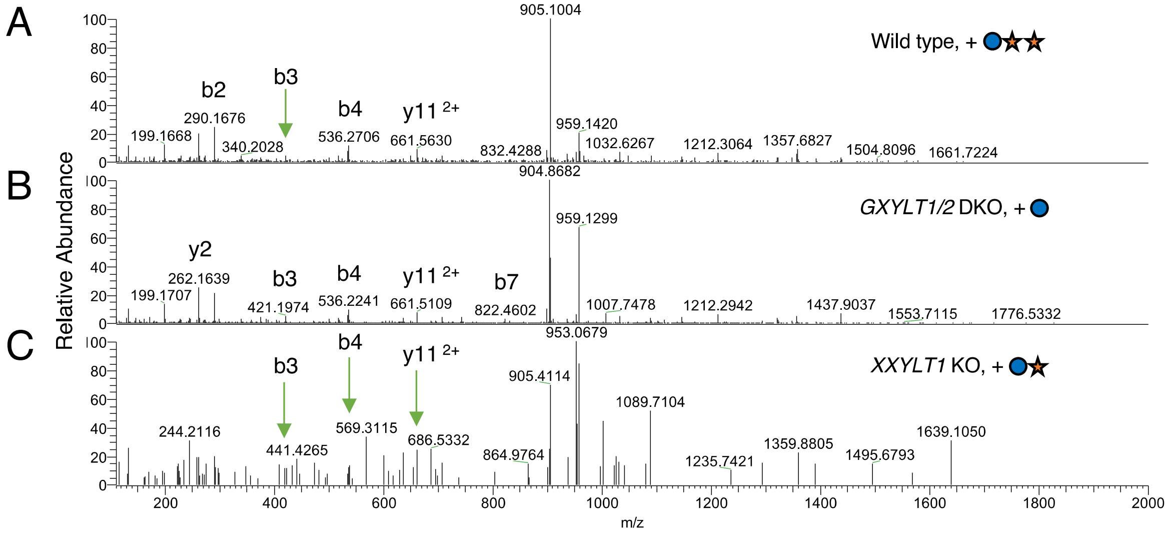

EGF13

Figure S2-4

488- G V H C E L E V N E C Q S N P C V N N G Q C V D K -512

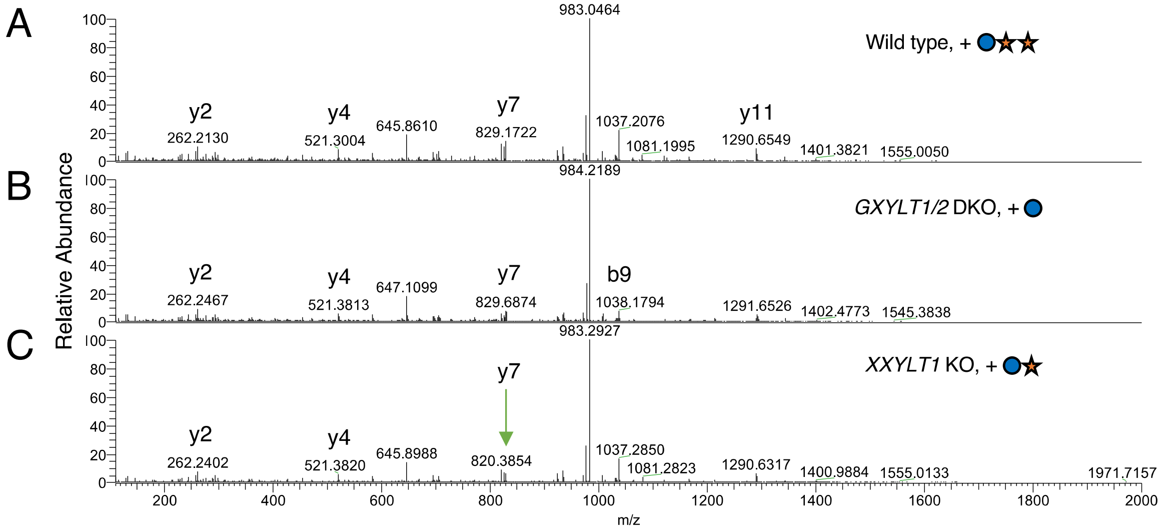

EGF14

Figure S2-5

516- F Q C L C P P G F T G P V C Q I D I D D C S S T P C L N G A K -546

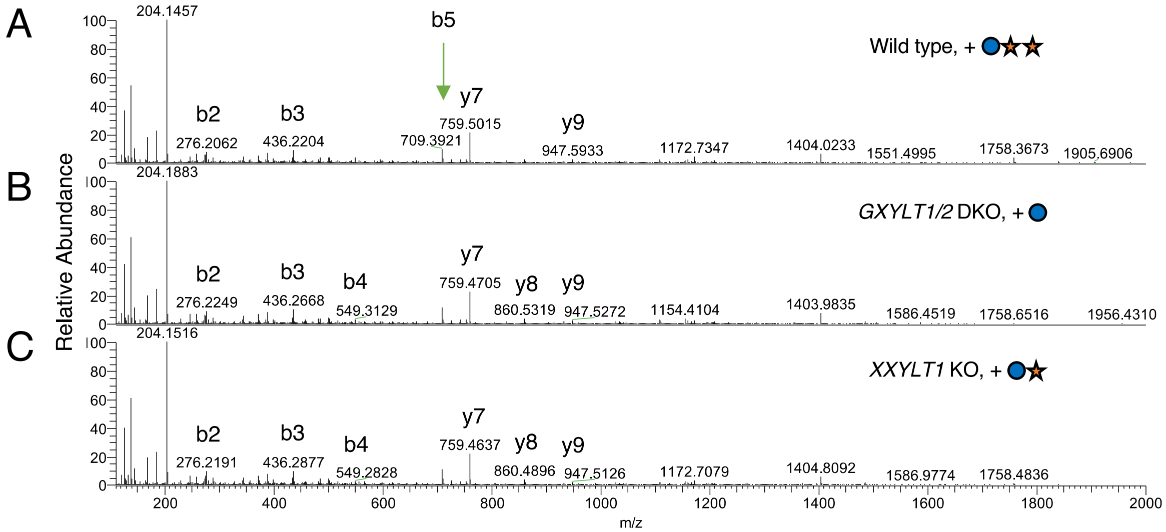

EGF16

Figure 2-6

611- C Y S S P C L N D -619

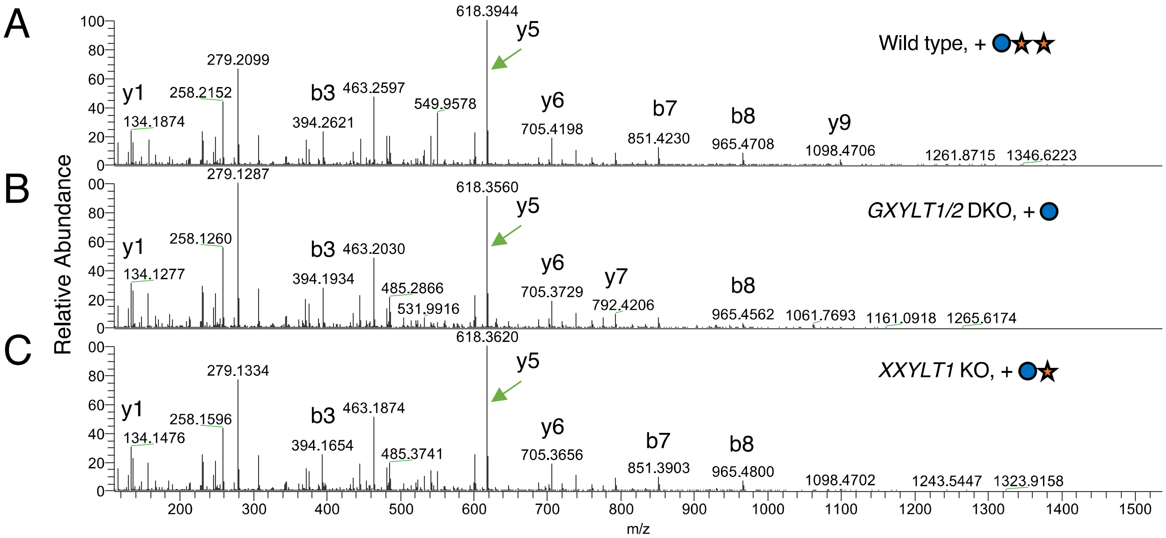

EGF18

Figure S2-7

679- C N I D I D E C A S N P C R -692

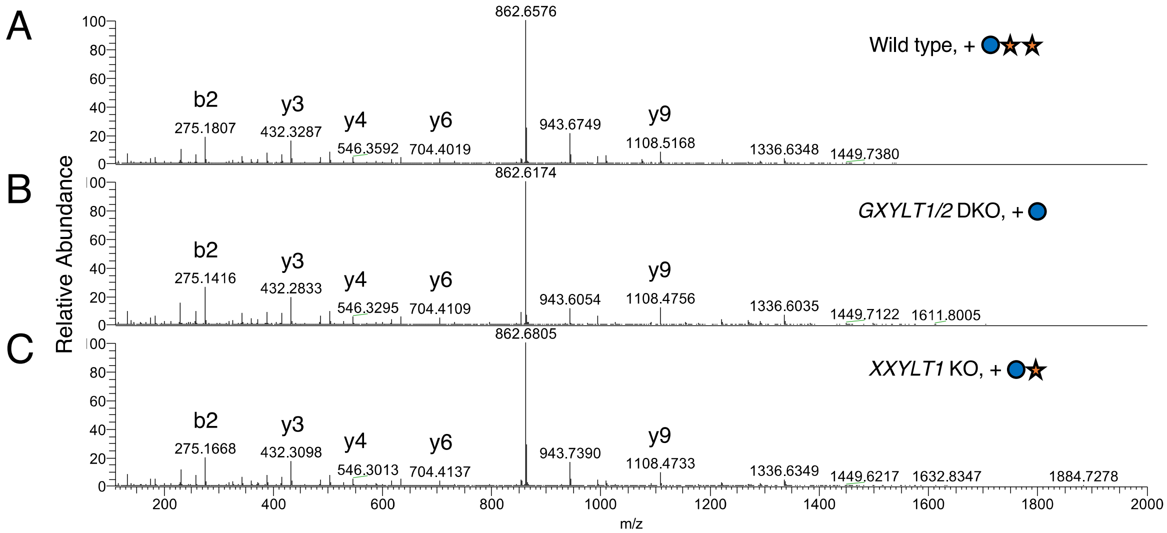

EGF19

Figure S2-8

706- C I C P E G P H H P S C Y S Q V N E C L S N P C I H G N C T G G L S G Y K -742

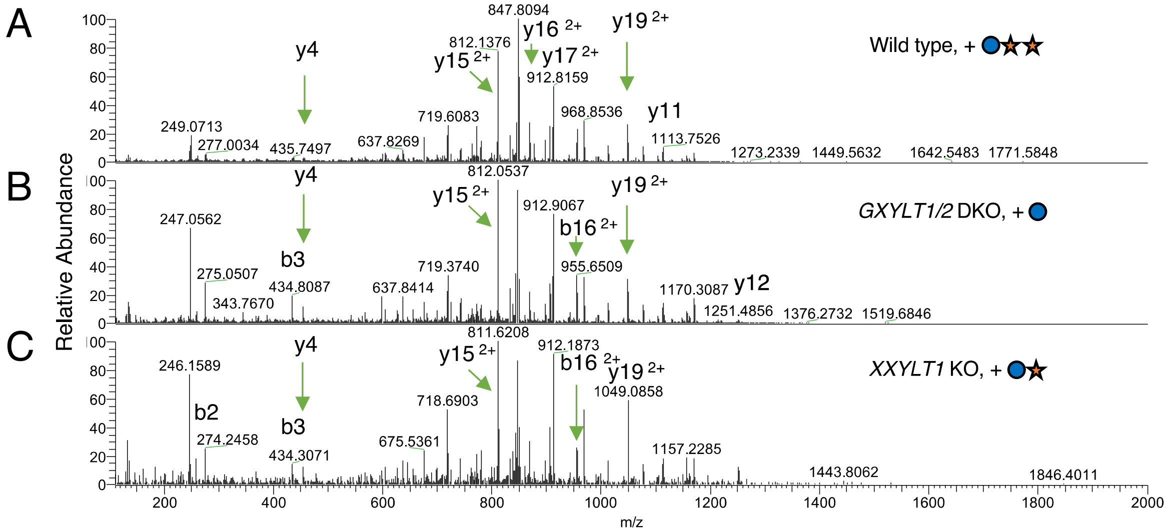

EGF20

Figure S2-9

759- N E C L S N P C Q N G G T C N N L V N G Y R -780

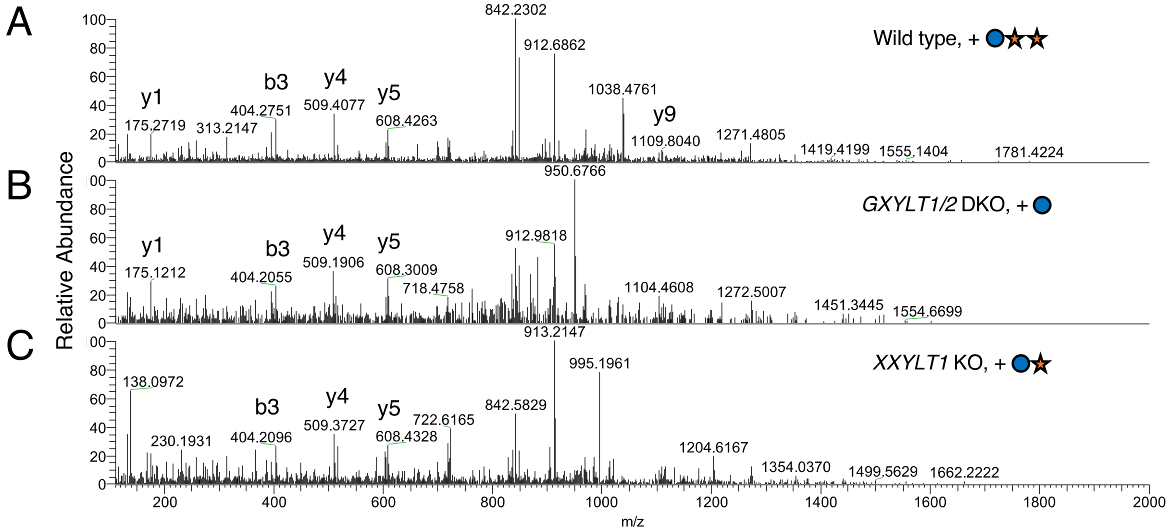

EGF21

Figure S2-10

789- G Y N C Q V N I D E C A S N P C L N Q G T C F D D V S G Y T C H C M L P Y T G K -828

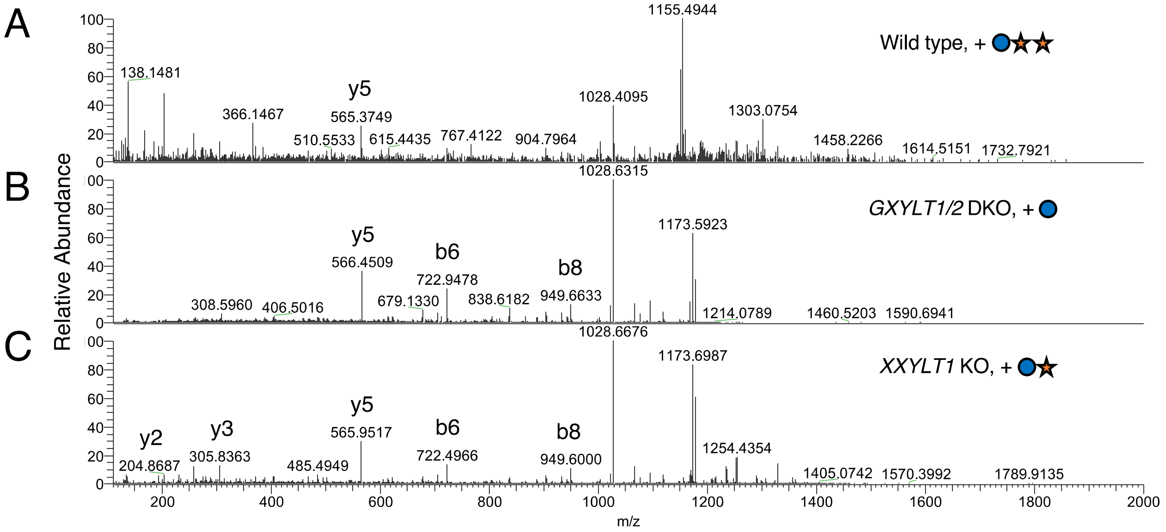

EGF23

Figure S2-11

870- C T V D V D E C I S K -880

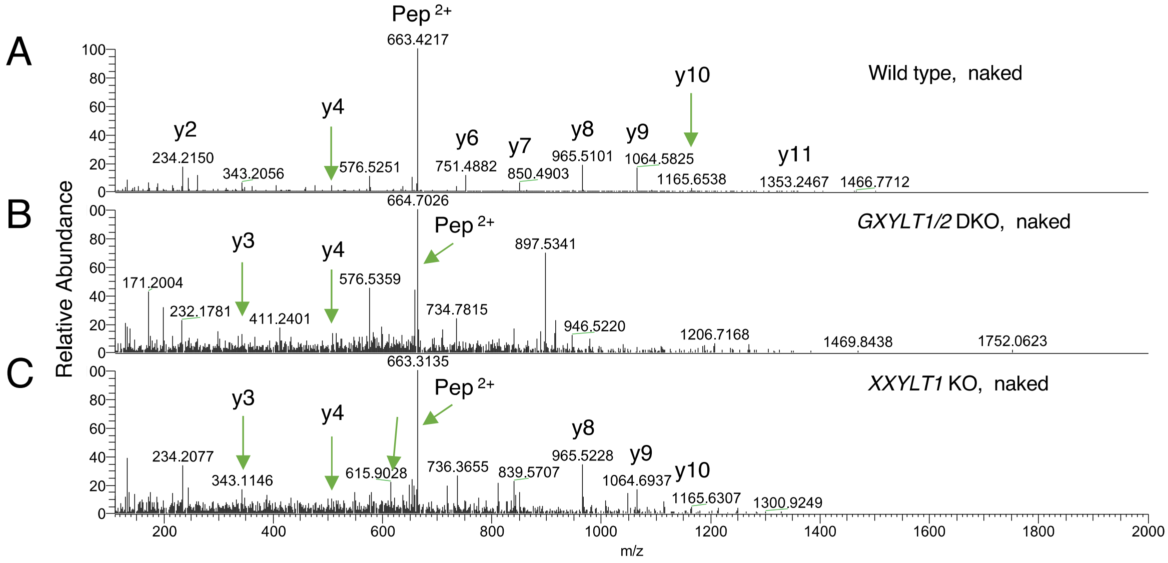

EGF25

Figure S2-12

946- C Q T D M N E C L S E P C K -959

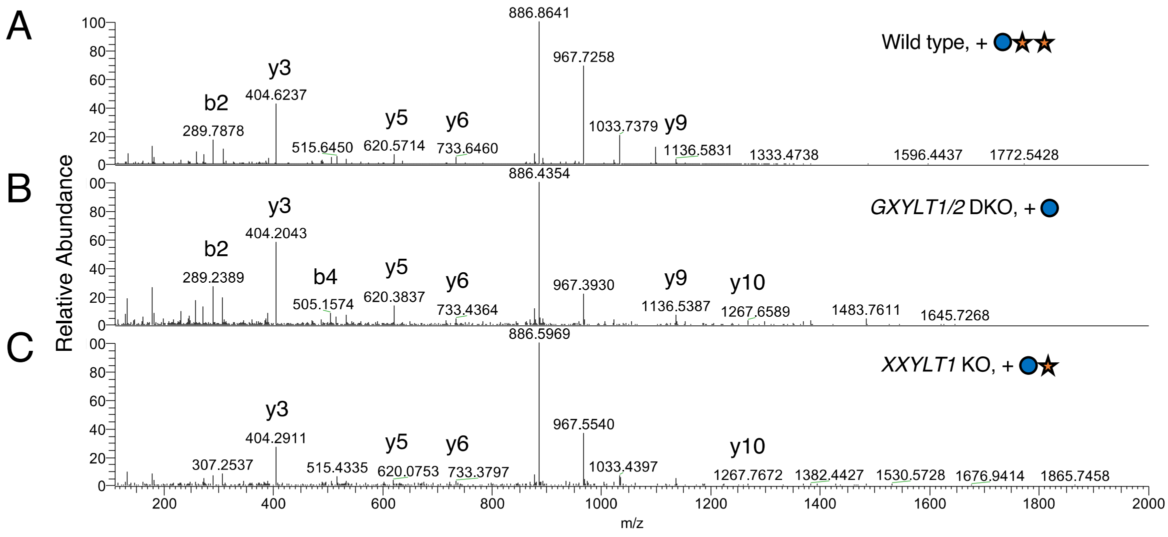

EGF27

Figure S2-13

1022- C L H D I N E C S S N P C L N A G T C V D G L G T Y H -1047

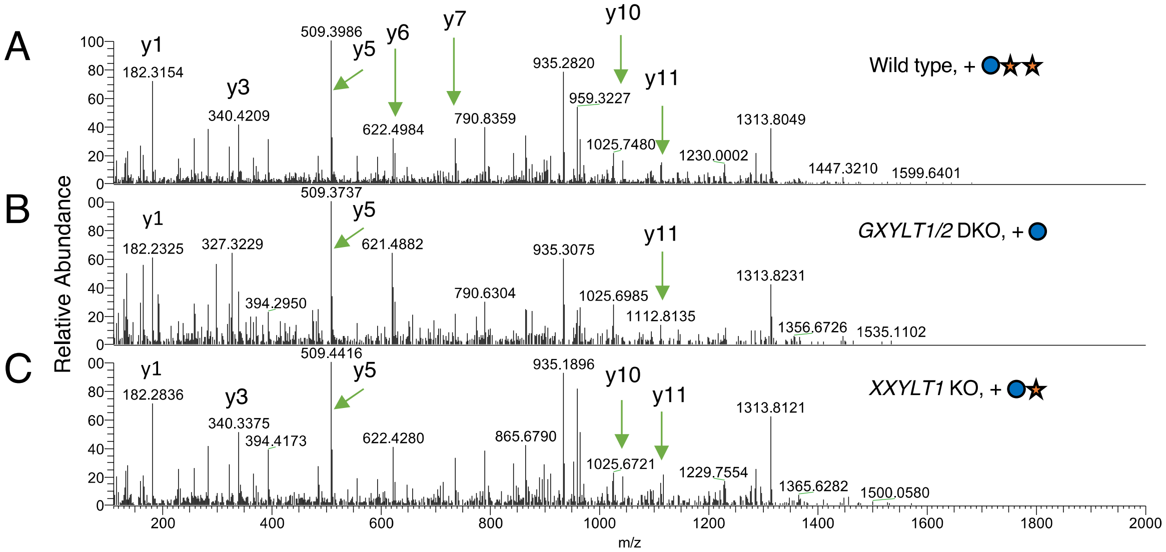

EGF30

Figure S2-14

1153- C A S N P C Q H G A T C N D F I G G Y R C E -1174

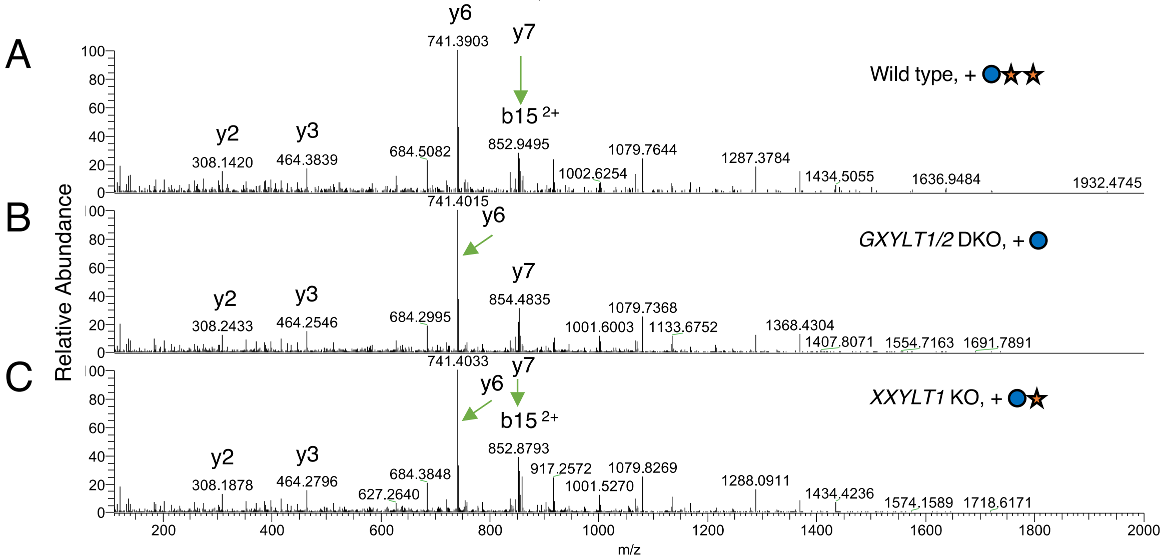

EGF33

Figure S2-15

1261- C E G D I N E C L S N P C S S E G S L D C V Q L K -1285

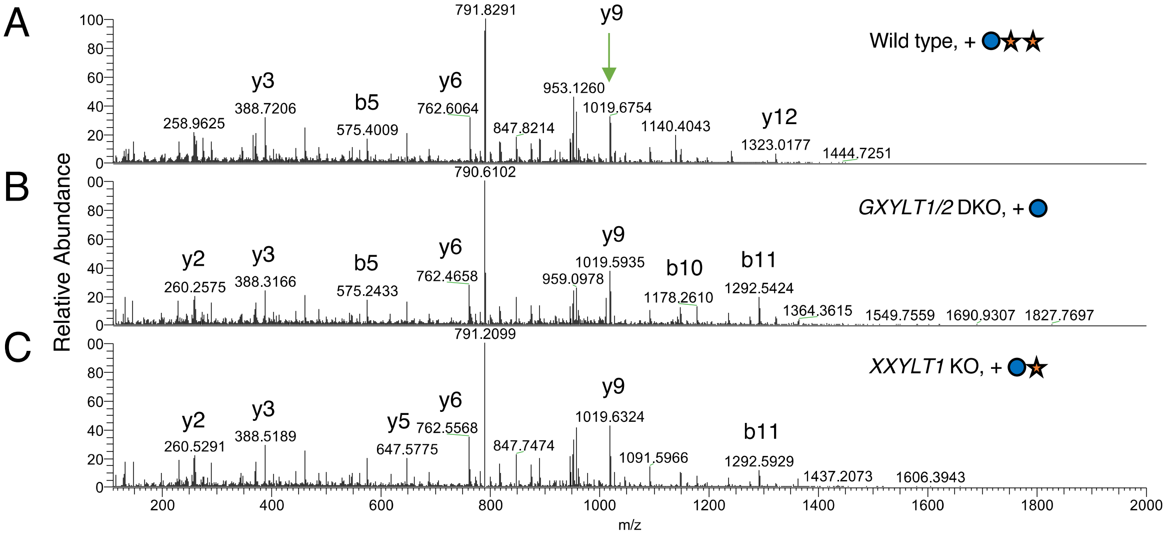

EGF36

Figure S2-16

57- C Q D S N P C L S T P C K -69

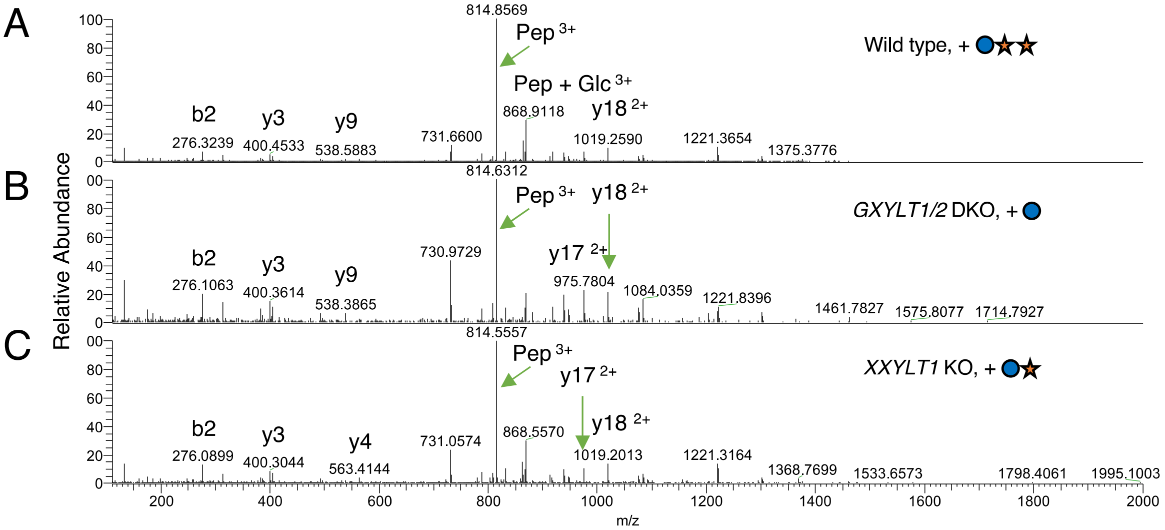

Figure S2. MS/MS spectra of (glyco)peptides from NOTCH2. MS/MS spectra of (glyco)peptide from NOTCH2. Samples were generated in wild type control HEK293T cells (A), *GXYLT1/2* DKO cells (B), *XXYLT1* KO cells (C) transfected with the plasmids encoding mouse NOTCH2 ECDs as described in Materials and Methods. The data in Figure S2-1 through S2-16 are derived from the analysis of mouse NOTCH2 EGF1-36. MS/MS spectra confirmed the identity of (glyco)peptides based on the presence of peptide specific b- and y- ions and neutral loss of predicted glycans. Amino acid sequences are shown with the identified fragment ions at the upper right corner. For each EGF repeat from NOTCH2, MS/MS spectra of glycopeptides modified with the major glycoform derived from each

cell clone are shown. The sequence of peptides, the predicted and measured m/z, and the charge state are summarized in Table S2.

A

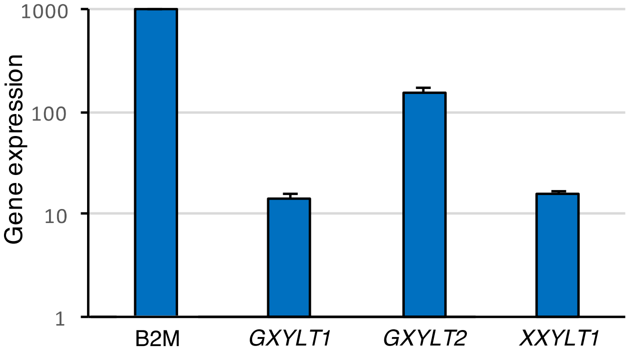

B

XXYLT1* KO (clone: 3C4): 10-bp deletion in *XXYLT1

```
Wild Type:CCTCGCCAAGTTCGAGGCGCACGAGGTGCTTAA
Allele  1:CCTCGCCA----------GCACGAGGTGCTTAA
```

GXYLT1/2* double KO (clone: 13D3, *GXYLT1* KO cell background): 7-bp deletion (allele 1) or 3-bp and 7-bp deletion (allele 2) in *GXYLT2

GXYLT1

```
Wild Type:GGCCCCGCAGCGCATCCCGGC------------GTGTCGGACAGgtacgggcag
Allele  1:GGCCCCGCAGCGCAT-------------------TGTCGGACAGgtacgggcag
Allele  2:GGCCCCGCAGCG-------------------------------------ggcag
Allele  3:--(76-bp-del)--------CTGCCCGTACCTGTGTCGGACAGgtacgggcag
```

GXYLT2

```
Wild Type:GCTGTGGTGGCCTGTGGCAATCGGCTGGAG
Allele  1:GCTGTGGTGGCCTGT-------GGCTGGAG
Allele  2:GCTG---TGGCCTGTGGCAATC-------G
```

Figure S3. *GXYLT1*, *GXYLT2*, and *XXYLT1* are expressed in HEK293T cells. (A) The RT-qPCR analysis confirmed the gene expression of *GXYLT1*, *GXYLT2*, and *XXYLT1* in HEK293T cells. (B) Genomic sequence of the edited genomic regions in *GXYLT1*, *GXYLT2*, and *XXYLT1* in wild type HEK293T cells and corresponding knockout clones.

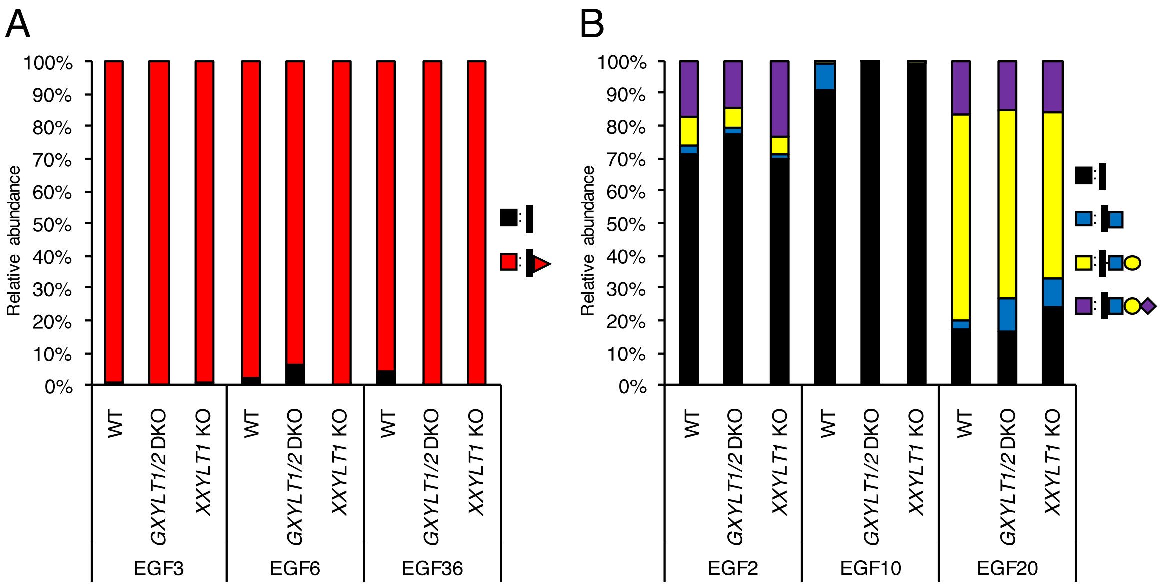

Figure S4. Knockout of the xylosyltransferases does not affect the attachment and elongation of *O*-fucose and *O*-GlcNAc glycans. EGF1-18, 19-36, and 24-28 from mouse NOTCH1 were produced in wild type, *GXYLT1/2* DKO, and *XXYLT1* KO HEK293T cells and purified from the medium as described in Materials and Methods. The protein was reduced, alkylated, purified by SDS-PAGE, and subjected to in-gel protease digestion. The resulting peptides were analyzed by LC-MS/MS, as described in Materials and Methods. (A) shows stoichiometry of *O*-fucose (Fuc) glycans on EGF3, EGF6 and EGF36 in NOTCH1, and (B) shows stoichiometry of *O*-GlcNAc glycans on EGF2, EGF10 and EGF20 in NOTCH1. Black bar, naked peptide; red triangle, Fuc; blue square, GlcNAc; yellow circle, galactose; purple diamond, Neu5Ac.

EGF3

Figure S5-1

113- N G G T C D L L T L T E Y K -126

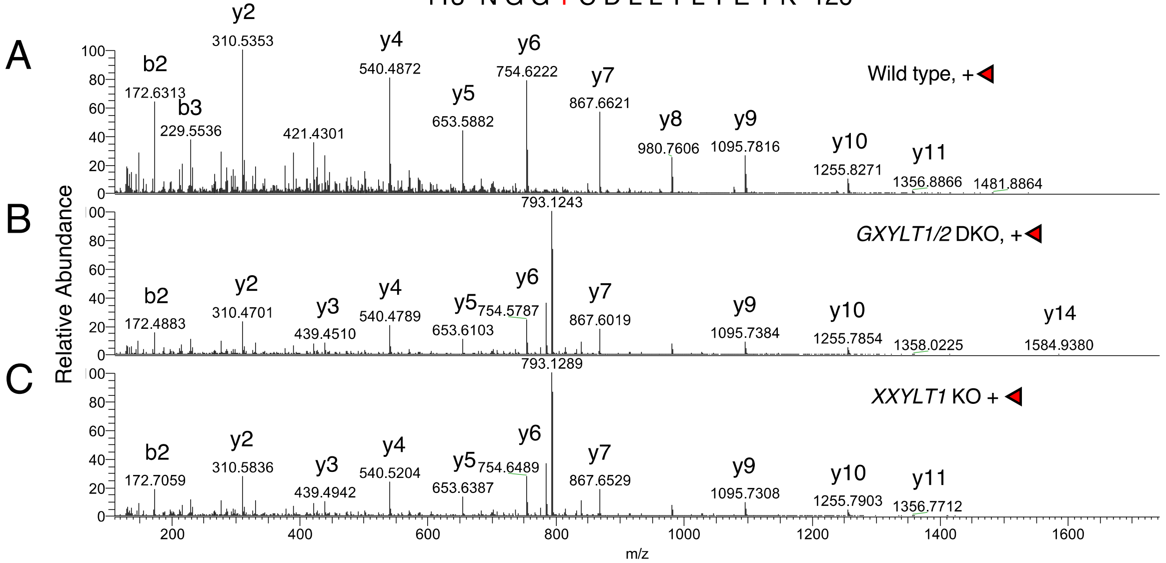

EGF6

Figure S5-2

208- A T H T G P H C E L P Y Y V P C S P S P C Q N G G T C R -234

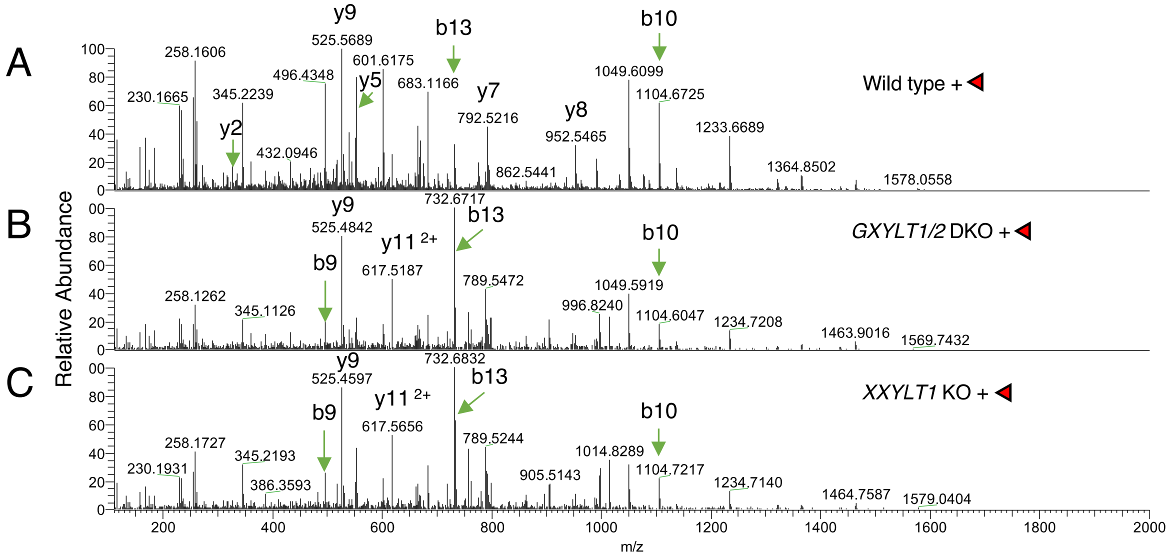

Figure S5-3

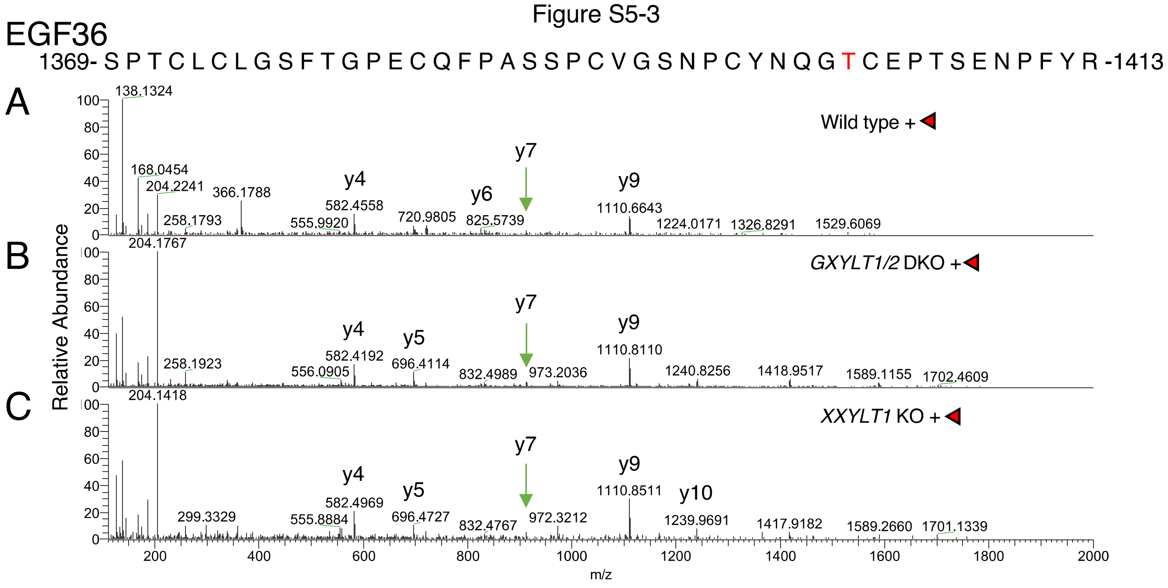

Figure S5. MS/MS spectra of (glyco)peptides with the predicted *O*-fucose site from NOTCH1. Samples were generated in wild type control HEK293T cells (A), *GXYLT1/2* DKO cells (B), *XXYLT1* KO cells (C) transfected with the plasmids encoding mouse NOTCH1 ECDs as described in Materials and Methods. The data in Figure S5-1 and S5-2 are derived from the analysis of mouse NOTCH1 EGF1-18. The data in Figure S5-3 are derived from the analysis of mouse NOTCH1 EGF19-36. MS/MS spectra confirmed the identity of (glyco)peptides based on the presence of peptide-specific b- and y-ions and neutral loss of predicted glycans. Amino acid sequences are shown with the identified fragment ions at the upper right corner. For each EGF repeat from NOTCH1, MS/MS spectra of glycopeptides modified with the major glycoform derived from each cell clone are shown. The sequence of peptides, the predicted and measured mass (m/z), and the charge state are summarized in Table S3.

Figure S6-1

EGF2

70- N A G T C H V V D H G G T V D Y A C S C P L G F **S** G P L C L T P L D N A C L A N P C R -112

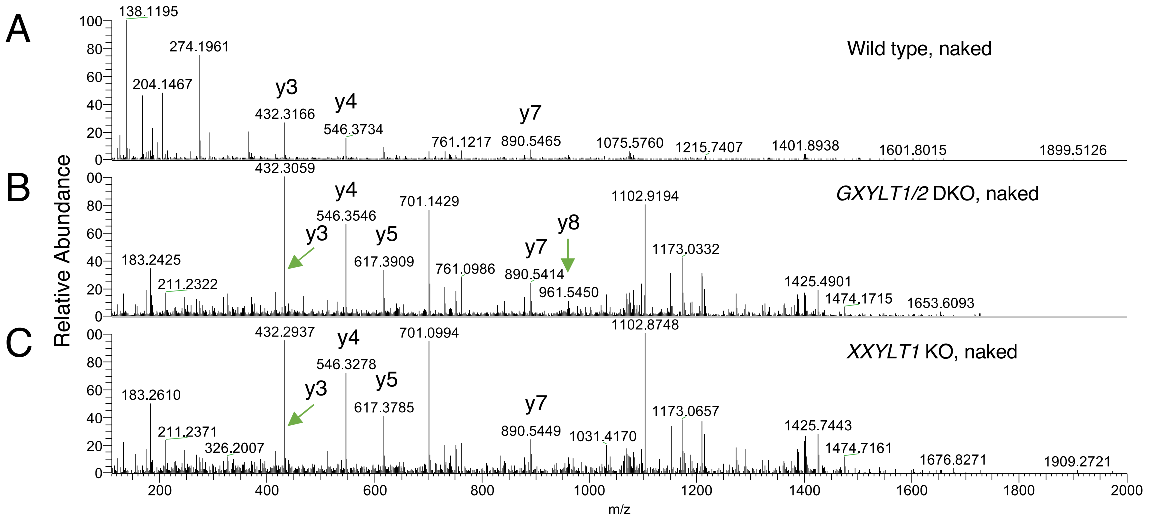

Figure S6-2

EGF10

396- A I C T C P S G Y **T** G P A C S Q D V D E C A L G A N P C E H A G K -428

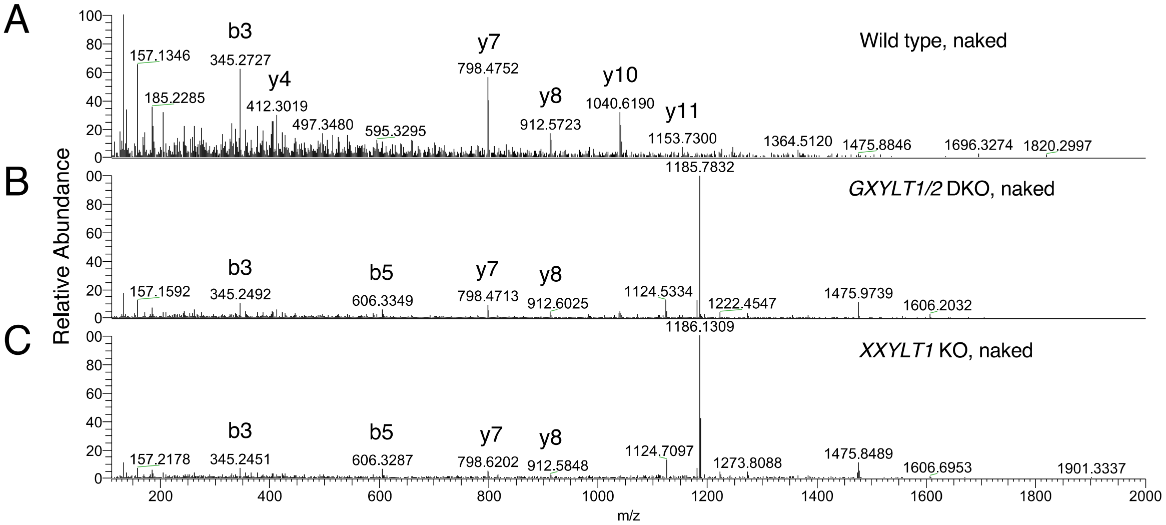

Figure S6-3

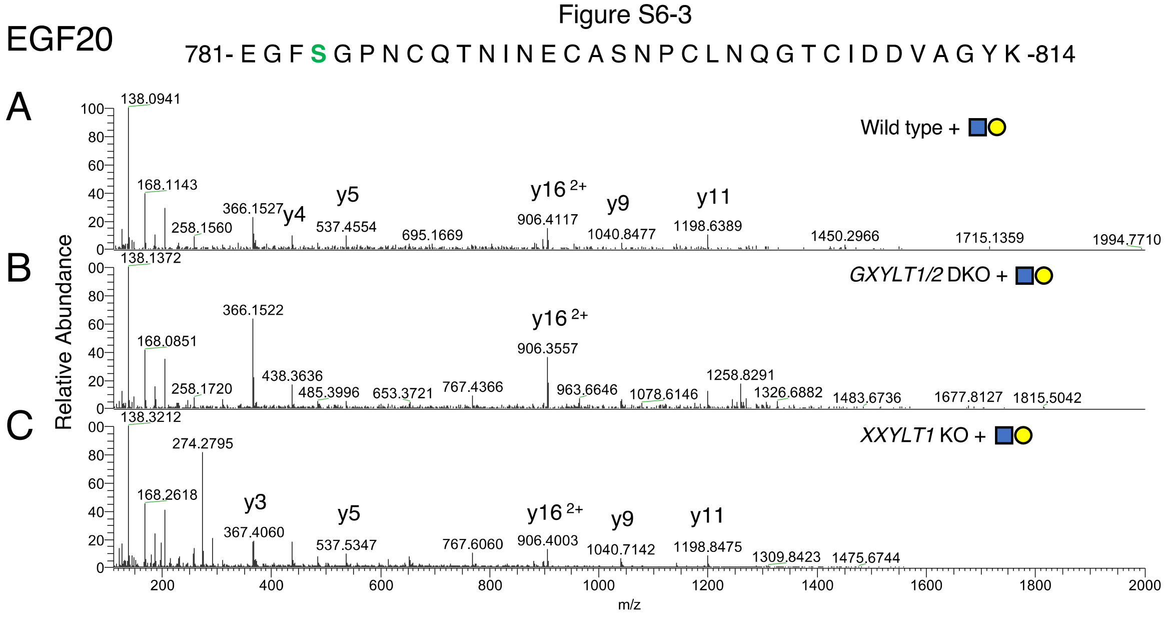

Figure S6. MS/MS spectra of (glyco)peptides with the predicted *O*-GlcNAc site from NOTCH1. Samples were generated in wild type control HEK293T cells (A), *GXYLT1/2* DKO cells (B), *XXYLT1* KO cells (C) transfected with the plasmids encoding mouse NOTCH1 ECDs as described in Materials and Methods. The data in Figure S6-1 and S6-2 are derived from the analysis of mouse NOTCH1 EGF1-18. The data in Figure S6-3 are derived from the analysis of mouse NOTCH1 EGF19-36. MS/MS spectra confirmed the identity of (glyco)peptides based on the presence of peptide-specific b- and y-ions and neutral loss of predicted glycans. Amino acid sequences are shown with the identified fragment ions at the upper right corner. For each EGF repeat from NOTCH1, MS/MS spectra of glycopeptides modified with the major glycoform derived from each cell clone are shown. The sequence of peptides, the predicted and measured mass (m/z), and the charge state are summarized in Table S4.

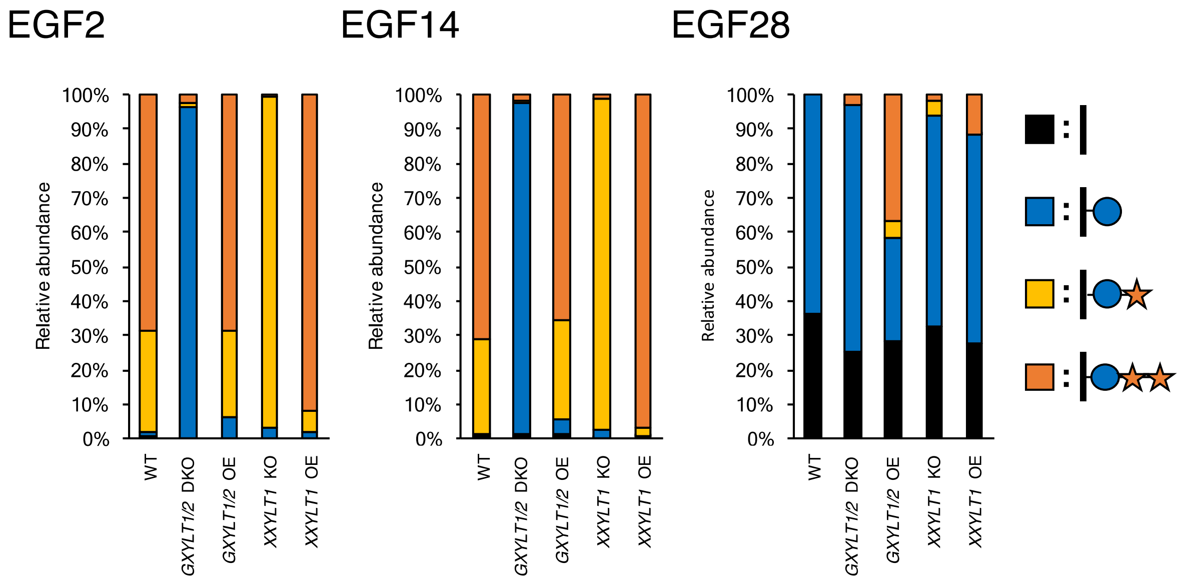

Figure S7. Co-transfection of *XYLT*s with NOTCH1 rescues xylosylation on NOTCH1. NOTCH1 EGF1-18 and 19-36 are co-transfected with GXYLT1 and GXYLT2 in *GXYLT1/2* DKO cells or with *XXYLT1* in *XXYLT1* KO cells. The Notch proteins are purified from the medium as in Materials and Methods. Mass spectral data on EGF2, 14, and 28 are shown. Black bar, naked peptide; blue circle, *O*-Glc; orange star, xylose.

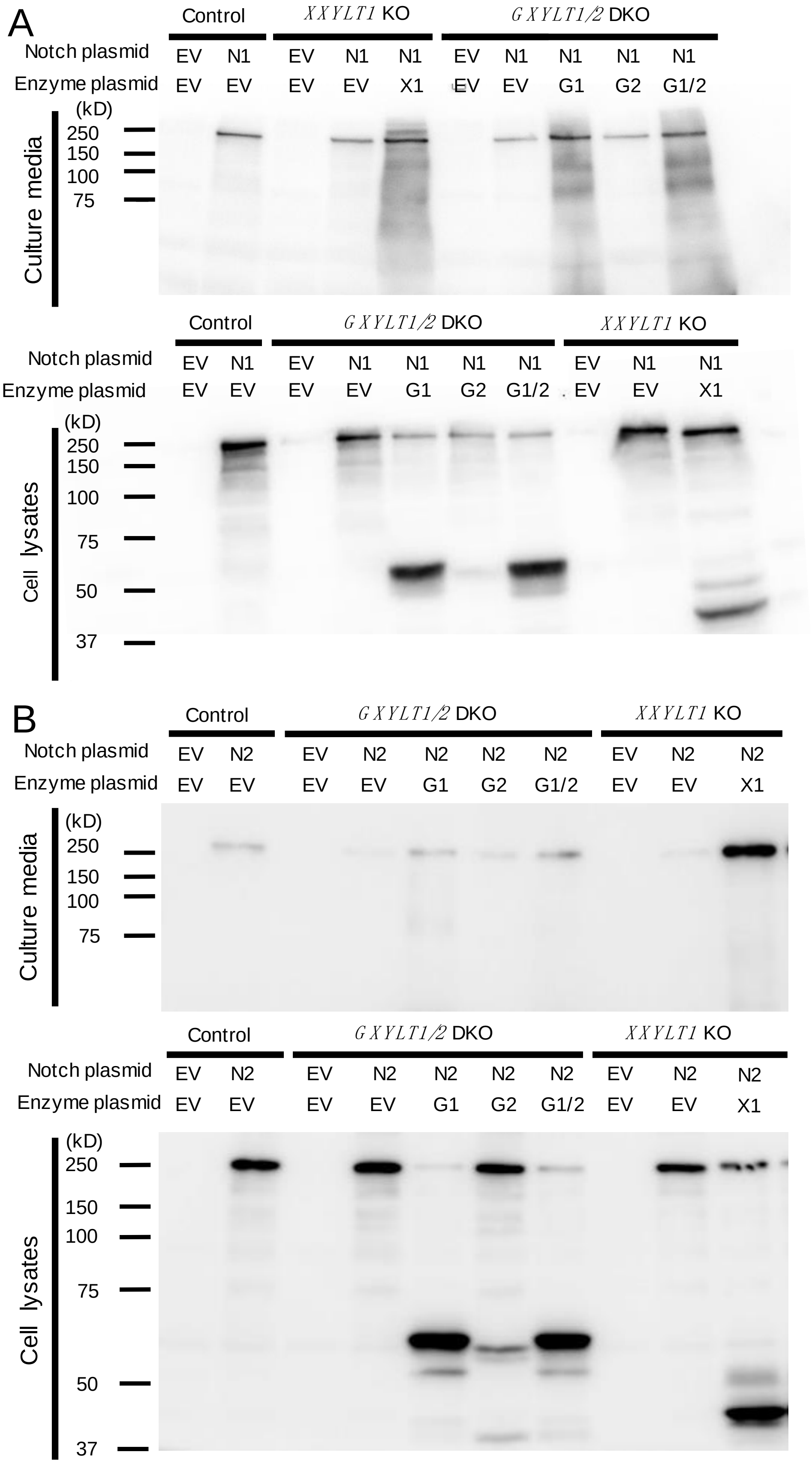
A
Control
XXYLT1 KO
GXYLT1/2 DKO
Notch plasmid EV N1 EV N1 N1 EV N1 N1 N1 N1
Enzyme plasmid EV EV EV EV X1 EV EV G1 G2 G1/2
(kD)
250
150
100
75
Culture media
Control
GXYLT1/2 DKO
XXYLT1 KO
Notch plasmid EV N1 EV N1 N1 N1 N1 EV N1 N1
Enzyme plasmid EV EV EV EV G1 G2 G1/2 EV EV X1
(kD)
250
150
100
75
50
37
Cell lysates
B
Control
GXYLT1/2 DKO
XXYLT1 KO
Notch plasmid EV N2 EV N2 N2 N2 N2 EV N2 N2
Enzyme plasmid EV EV EV EV G1 G2 G1/2 EV EV X1
(kD)
250
150
100
75
Culture media
Control
GXYLT1/2 DKO
XXYLT1 KO
Notch plasmid EV N2 EV N2 N2 N2 N2 EV N2 N2
Enzyme plasmid EV EV EV EV G1 G2 G1/2 EV EV X1
(kD)
250
150
100
75
50
37
Cell lysates

Figure 8. The raw data for Figure 6 "Xylosyl-extension of *O*-Glc glycans enhances the secretion of the ECDs of NOTCH1 and NOTCH2 overexpressed in HEK293T cells." (A) Data for Figure 6A. Secretion assay with the Myc-His6-tagged version of the EGF1-36 of NOTCH1 (N1 EGF1-36) in the wild type control and *XYLTs*' KO clones. The N1 EGF1-36 proteins in the culture media and the cell lysates were detected by western blotting using an anti-Myc antibody. EV, empty vector; N1, NOTCH1; G1, GXYLT1; G2; GXYLT2, X1; XXYLT1. (B) Data for Figure 6C. Secretion assay with the Myc-His6-tagged version of the EGF1-36 of NOTCH2 (N2 EGF1-36) in the wild type control and *XYLTs*' KO clones. The N2 EGF1-36 proteins in the culture media and the cell lysates were detected by western blotting using an anti-Myc antibody. EV, empty vector; N1, NOTCH1; G1, GXYLT1; G2; GXYLT2, X1; XXYLT1.

Table S1. Summary of mass spectral semi-quantification of NOTCH1 *O*-Glc glycans. The number of EGF repeats, the number of amino acids counted from the N-terminal, the peptide sequences, predicted mass (m/z), charge state, glycoform, measured mass (m/z), and glycoform ratio of the analyzed peptides are shown. In the glycoform ratio column, the average height of EICs and the standard error of the mean are shown (N=3). *Two biological replicates (N=2). The average and the range of two measurements are shown in the glycoform ratio column. Colored letters in sequences indicate post-translational modification sites. Blue, *O*-Glc; red, *O*-Fuc; green, *O*-GlcNAc.

						WT		*GXYLT1/2* DKO		*XXYLT1* KO	
EGF	A.A.	Sequence	Predicted mass (m/z)	charge state	Glycoform	measured mass (m/z)	Glycoform Ratio	measured mass (m/z)	Glycoform Ratio	measured mass (m/z)	Glycofor m Ratio
2	57-69	CQDSNPCLSTPCK	775.3081	2+	naked	775.3086	1 ± 0 %	775.3055	1 ± 0 %	775.3214	0 ± 0 %
2	57-69	CQDSNPCLSTPCK	856.3345	2+	Glc	856.3346	1 ± 0 %	856.3477	98 ± 1 %	856.4729	2 ± 0 %
2	57-69	CQDSNPCLSTPCK	922.3557	2+	Xyl-Glc	933.3562	29 ± 3 %	922.2513	0 ± 0 %	933.1764	97 ± 0 %
2	57-69	CQDSNPCLSTPCK	988.3769	2+	Xyl-Xyl-Glc	988.3775	69 ± 3 %	988.3156	1 ± 1 %	988.8435	0 ± 0 %
4	137-165	SCQQADPCASNPCANGGQCLPFESSYICR	1112.1200	3+	naked	ND	0 ± 0 %	ND	0 ± 0 %	ND	0 ± 0 %
4	137-165	SCQQADPCASNPCANGGQCLPFESSYICR	1166.1376	3+	Glc	1166.807	3 ± 0 %	1166.0687	100 ± 0 %	1166.0870	4 ± 1 %
4	137-165	SCQQADPCASNPCANGGQCLPFESSYICR	1210.1517	3+	Xyl-Glc	1210.821	15 ± 2 %	1210.6472	0 ± 0%	1210.9284	95 ± 1 %
4	137-165	SCQQADPCASNPCANGGQCLPFESSYICR	1254.1658	3+	Xyl-Xyl-Glc	1254.833	82 ± 2 %	1254.3231	0 ± 0 %	1254.2387	0 ± 0 %
9	328-345	TGEDCSENIDDCASAACF	1011.3620	2+	naked	1011.3359	100 ± 0 %	1011.4922	100 ± 0 %	1011.2375*	100 ± 0 %
9	328-345	TGEDCSENIDDCASAACF	1092.3884	2+	Glc	ND	0 ± 0 %	ND	0 ± 0 %	ND*	0 ± 0 %
9	328-345	TGEDCSENIDDCASAACF	1158.4096	2+	Xyl-Glc	ND	0 ± 0 %	ND	0 ± 0 %	ND*	0 ± 0 %
9	328-345	TGEDCSENIDDCASAACF	1224.4307	2+	Xyl-Xyl-Glc	ND	0 ± 0 %	ND	0 ± 0 %	ND*	0 ± 0 %
10	366-395	TGLLCHLNDACISNPCNEGSNCDTNPVNGK	1111.479	3+	naked	1111.285	0 ± 0 %	1111.7050	1 ± 0 %	1111.7465	0 ± 0 %
10	366-395	TGLLCHLNDACISNPCNEGSNCDTNPVNGK	1165.4966	3+	Glc	1165.825	11 ± 1 %	1165.7761	24 ± 2 %	1165.4897	4 ± 2 %
10	366-395	TGLLCHLNDACISNPCNEGSNCDTNPVNGK	1209.5107	3+	Xyl-Glc	1209.844	9 ± 0 %	1209.4076	0 ± 0 %	1209.5458	43 ± 4 %
10	366-395	TGLLCHLNDACISNPCNEGSNCDTNPVNGK	1253.5248	3+	Xyl-Xyl-Glc	1253.857	28 ± 2 %	1253.2766	0 ± 0 %	1253.6475	0 ± 0 %
10	366-395	TGLLCHLNDACISNPCNEGSNCDTNPVNGK	1219.5142	3+	Hex-Glc	1219.846	24 ± 1 %	1219.1594	29 ± 4 %	1219.1119	13 ± 2 %
10	366-395	TGLLCHLNDACISNPCNEGSNCDTNPVNGK	1316.546	3+	Neu5Ac-Hex-Glc	1316.879	27 ± 1 %	1316.4708	47 ± 4 %	1316.2974	39 ± 2 %
12	445-474	TGPRCEIDVNECISNPCQNDATCLDQIGEF	1220.5197	3+	naked	ND	0 ± 0 %	1220.4792	0 ± 0 %	ND*	0 ± 0 %
12	445-474	TGPRCEIDVNECISNPCQNDATCLDQIGEF	1274.5373	3+	Glc	1274.5638	0 ± 0 %	1274.0725	55 ± 6 %	1274.5297*	4 ± 3 %
12	445-474	TGPRCEIDVNECISNPCQNDATCLDQIGEF	1318.5514	3+	Xyl-Glc	1318.4622	2 ± 2 %	1318.3816	1 ± 0 %	1318.0804*	96 ± 4 %
12	445-474	TGPRCEIDVNECISNPCQNDATCLDQIGEF	1362.5655	3+	Xyl-Xyl-Glc	1362.1283	98 ± 2 %	1362.6799	0 ± 0 %	1362.4866*	0 ± 0 %
12	445-474	TGPRCEIDVNECISNPCQNDATCLDQIGEF	1328.5549	3+	Hex-Glc	1328.7328	0 ± 0 %	1328.4028	31 ± 3 %	1328.9224*	0 ± 0 %
12	445-474	TGPRCEIDVNECISNPCQNDATCLDQIGEF	1425.5867	3+	Neu5Ac-Hex-Glc	ND	0 ± 0 %	1425.9257	13 ± 6 %	1425.9411*	0 ± 0 %
13	482-512	CEINTDECASSPCLHNGHCMDKINEF	785.5687	4+	naked	785.2771	0 ± 0 %	785.0084	0 ± 0 %	785.0961*	0 ± 0 %
13	482-512	CEINTDECASSPCLHNGHCMDKINEF	826.0819	4+	Glc	826.5149	0 ± 0 %	826.3158	100 ± 0 %	826.3374*	17 ± 13 %
13	482-512	CEINTDECASSPCLHNGHCMDKINEF	859.0924	4+	Xyl-Glc	858.8657	1 ± 1 %	858.4297	0 ± 0 %	858.1957*	83 ± 13 %
13	482-512	CEINTDECASSPCLHNGHCMDKINEF	892.1030	4+	Xyl-Xyl-Glc	892.1840	99 ± 1 %	892.3415	0 ± 0 %	892.2477*	0 ± 0 %

14	519-538	GFNGHLCQYDVDECASTPCK	786.6582	3+	naked	ND	1 ± 0 %	786.9038	1 ± 0 %	786.4928	± 0 %
14	519-538	GFNGHLCQYDVDECASTPCK	840.6758	3+	Glc	ND	1 ± 0 %	840.1834	98 ± 1 %	840.6792	2 ± 0 %
14	519-538	GFNGHLCQYDVDECASTPCK	884.6899	3+	Xyl-Glc	885.0549	28 ± 5 %	884.1941	1 ± 0 %	884.6925	97 ± 1 %
14	519-538	GFNGHLCQYDVDECASTPCK	928.704	3+	Xyl-Xyl-Glc	929.0391	71 ± 5 %	928.5175	1 ± 1 %	928.1634	0 ± 0 %
16	582-613	DGVATFTCLCQPGYTGHHCETNINECHSQPCR	1007.16418	4+	naked	ND	0 ± 0 %	ND	0 ± 0 %	ND	0 ± 0 %
16	582-613	DGVATFTCLCQPGYTGHHCETNINECHSQPCR	1047.67738	4+	Glc	ND	0 ± 0 %	1047.3729	100 ± 0 %	ND	0 ± 0 %
16	582-613	DGVATFTCLCQPGYTGHHCETNINECHSQPCR	1080.68795	4+	Xyl-Glc	ND	0 ± 0 %	ND	0 ± 0 %	1080.2937	100 ± 0 %
16	582-613	DGVATFTCLCQPGYTGHHCETNINECHSQPCR	1113.69853	4+	Xyl-Xyl-Glc	1114.2009	100 ± 0 %	ND	0 ± 0 %	ND	0 ± 0 %
17	632-658	GTTGPNCEINLDDCASNPCDSGTCLDK	1112.77643	3+	naked	1111.4284	1 ± 0 %	1112.2847	28 ± 3 %	1112.4478	1 ± 1 %
17	632-658	GTTGPNCEINLDDCASNPCDSGTCLDK	1166.79403	3+	Glc	1166.8013	1 ± 0 %	1166.5844	68 ± 3 %	1166.7941	1 ± 1 %
17	632-658	GTTGPNCEINLDDCASNPCDSGTCLDK	1210.80813	3+	Xyl-Glc	1211.1553	29 ± 3 %	1211.3663	2 ± 1 %	1211.4894	97 ± 1 %
17	632-658	GTTGPNCEINLDDCASNPCDSGTCLDK	1254.82223	3+	Xyl-Xyl-Glc	1254.8311	69 ± 3 %	1254.1699	2 ± 0 %	1254.6852	1 ± 1 %
19	704-731	CPEGYHDPTCLSEVNECNSNPCIHGACR	834.0869	3+	naked	834.6639	29 ± 1 %	834.9720	24 ± 5 %	834.0064	33 ± 2 %
19	704-731	CPEGYHDPTCLSEVNECNSNPCIHGACR	874.6001	3+	Glc	874.6599	4 ± 1 %	874.5538	75 ± 5 %	874.2295	3 ± 1 %
19	704-731	CPEGYHDPTCLSEVNECNSNPCIHGACR	907.6106	3+	Xyl-Glc	907.9304	1 ± 1 %	907.0809	0 ± 0 %	907.7001	64 ± 2 %
19	704-731	CPEGYHDPTCLSEVNECNSNPCIHGACR	940.6212	3+	Xyl-Xyl-Glc	940.8922	66 ± 3 %	940.7333	1 ± 1 %	940.5528	0 ± 0 %
20	739-769	CDCAPGWSGTNCDINNNECESNPCVNGGTCK	1232.1293	3+	naked	1232.0637	14 ± 2 %	1232.8530	13 ± 3 %	1232.0841	19 ± 2 %
20	739-769	CDCAPGWSGTNCDINNNECESNPCVNGGTCK	1286.1469	3+	Glc	1286.7441	2 ± 1 %	1286.4574	86 ± 3 %	1286.5417	2 ± 1 %
20	739-769	CDCAPGWSGTNCDINNNECESNPCVNGGTCK	1330.161	3+	Xyl-Glc	1329.7700	1 ± 1 %	1329.8492	1 ± 0 %	1330.5839	79 ± 1 %
20	739-769	CDCAPGWSGTNCDINNNECESNPCVNGGTCK	1374.1751	3+	Xyl-Xyl-Glc	1374.7952	84 ± 2 %	1374.6488	1 ± 1 %	1374.9282	0 ± 0 %
21	781-814	EGFSGPNCQTNINECASNPCLNQGTCIDDVAGYK	1076.4501	4+	naked	1076.9028	3 ± 1 %	1076.2213	32 ± 10 %	1076.6600	8 ± 6 %
21	781-814	EGFSGPNCQTNINECASNPCLNQGTCIDDVAGYK	1116.9633	4+	Glc	1116.1684	0 ± 0 %	1116.8744	65 ± 6 %	1116.4807	2 ± 1 %
21	781-814	EGFSGPNCQTNINECASNPCLNQGTCIDDVAGYK	1149.97388	4+	Xyl-Glc	1149.1235	21 ± 10 %	1150.7433	0 ± 0 %	1149.3826	88 ± 5 %
21	781-814	EGFSGPNCQTNINECASNPCLNQGTCIDDVAGYK	1182.98445	4+	Xyl-Xyl-Glc	1182.7345	76 ± 11 %	1182.1546	4 ± 4 %	1182.4293	2 ± 1 %
25	942-967	CEEDINECASNPCQNGADCTDCVDSY	1034.02793	3+	naked	1033.8633*	30 ± 13 %	1033.0846*	25 ± 4 %	1033.7456*	64 ± 0 %
25	942-967	CEEDINECASNPCQNGADCTDCVDSY	1088.04553	3+	Glc	1087.8568*	0 ± 0 %	1088.2751*	75 ± 4 %	1088.5379*	0 ± 0 %
25	942-967	CEEDINECASNPCQNGADCTDCVDSY	1132.05963	3+	Xyl-Glc	1132.7153*	1 ± 1 %	1132.7887*	0 ± 0 %	1132.8135*	36 ± 0 %
25	942-967	CEEDINECASNPCQNGADCTDCVDSY	1176.07373	3+	Xyl-Xyl-Glc	1176.6862*	69 ± 12 %	1176.9320*	0 ± 0 %	1176.1529*	0 ± 0 %
27	1021-1043	DVNECDSRPCLHGGTCQDSYGTY	952.0408	3+	naked	952.0937	45 ± 11 %	952.6852	30 ± 4 %	952.2100*	20 ± 16 %
27	1021-1043	DVNECDSRPCLHGGTCQDSYGTY	1006.0584	3+	Glc	1006.1933	49 ± 15 %	1006.1989	70 ± 4 %	1006.7749*	78 ± 16 %
27	1021-1043	DVNECDSRPCLHGGTCQDSYGTY	1050.0725	3+	Xyl-Glc	ND	0 ± 0 %	ND	0 ± 0 %	1050.6274*	2 ± 0 %
27	1021-1043	DVNECDSRPCLHGGTCQDSYGTY	1094.0866	3+	Xyl-Xyl-Glc	1094.5572	6 ± 4 %	ND	0 ± 0 %	ND*	0 ± 0 %
28	1062-1069	WCDSAPCK	512.2053	2+	naked	512.4991	36 ± 1 %	512.3639	37 ± 5 %	512.4764	41 ± 4 %
28	1062-1069	WCDSAPCK	593.2317	2+	Glc	593.7694	64 ± 1 %	593.4158	63 ± 5 %	593.2895	57 ± 4 %
28	1062-1069	WCDSAPCK	659.25285	2+	Xyl-Glc	660.4237	0 ± 0 %	659.4829	0 ± 0 %	659.1278	2 ± 1 %
28	1062-1069	WCDSAPCK	703.26695	2+	Xyl-Xyl-Glc	703.8407	0 ± 0 %	703.1400	0 ± 0 %	ND	0 ± 0 %
31	1176-1205	HGSDCSEEINECLSQPCQNGGTCIDLTNSY	1198.1480	3+	naked	1198.3647*	29 ± 3 %	1198.6564	20 ± 2 %	1198.0699*	0 ± 0 %
31	1176-1205	HGSDCSEEINECLSQPCQNGGTCIDLTNSY	1252.1656	3+	Glc	ND*	0 ± 0 %	1253.2573	80 ± 2 %	1252.7629*	1 ± 0 %

31	1176-1205	HGSDCSEEINECLSQPCQNGGTCIDLTNSY	1296.1797	3+	Xyl-Glc	ND*	0 ± 0 %	1296.4331	0 ± 0 %	1296.7633*	97 ± 1 %
31	1176-1205	HGSDCSEEINECLSQPCQNGGTCIDLTNSY	1340.1938	3+	Xyl-Xyl-Glc	1340.5931*	71 ± 3 %	1340.8524	0 ± 0 %	1340.8941*	2 ± 1 %
33	1264-1279	CEGDVNECLSNPCDPR	952.864525	2+	naked	952.1117	33 ± 4 %	952.8756	31 ± 4 %	952.3445	38 ± 2 %
33	1264-1279	CEGDVNECLSNPCDPR	1033.89093	2+	Glc	1033.2603	6 ± 0 %	1033.2455	68 ± 5 %	1033.2875	4 ± 1 %
33	1264-1279	CEGDVNECLSNPCDPR	1100.41208	2+	Xyl-Glc	1100.8854	25 ± 5 %	1100.3941	1 ± 0 %	1100.6274	58 ± 2 %

Table S2. Summary of mass spectral semi-quantification of NOTCH2 *O*-Glc glycans. The number of EGF repeats, the number of amino acids counted from the N-terminal, the peptide sequences, predicted mass (m/z), charge state, glycoform, measured mass (m/z), and glycoform ratio of the analyzed peptides are shown. In the glycoform ratio column, the average height of EICs and the standard error of the mean are shown (N=3). * Two biological replicates (N=2). The average and the range of two measurements are shown in the glycoform ratio column. Colored letters in sequences indicate post-translational modification sites. Blue, *O*-Glc; red, *O*-Fuc; green, *O*-GlcNAc.

						WT		*GXYLT1/2* DKO		*XXYLT1* KO	
EGF	A.A.	Sequence	Predicted mass (m/z)	charge state	Glycoform	measured mass (m/z)	Glycoform Ratio	measured mass (m/z)	Glycoform Ratio	measured mass (m/z)	Glycoform Ratio
4	141-169	QCQWTDACLSHPCENGSTCTSVASQFSCK	1184.8130	3+	naked	1184.2549	0 ± 0 %	1184.3248	0 ± 0 %	1184.2576	0 ± 0 %
4	141-169	QCQWTDACLSHPCENGSTCTSVASQFSCK	1238.8306	3+	Glc	1238.7652	40 ± 3 %	1238.8564	100 ± 0 %	1238.4761	26 ± 8 %
4	141-169	QCQWTDACLSHPCENGSTCTSVASQFSCK	1282.8447	3+	Xyl-Glc	1282.4973	59 ± 3 %	1282.7761	0 ± 0 %	1282.8400	72 ± 8 %
4	141-169	QCQWTDACLSHPCENGSTCTSVASQFSCK	1326.8588	3+	Xyl-Xyl-Glc	ND	0 ± 0 %	ND	0 ± 0 %	ND	0 ± 0 %
10	369-389	AGLLCHLDDACISNPCHK	521.2407	4+	naked	521.2441	0 ± 0 %	521.7644	1 ± 1 %	521.9007	0 ± 0 %
10	369-389	AGLLCHLDDACISNPCHK	561.7539	4+	Glc	561.8347	61 ± 10 %	561.3817	99 ± 1 %	561.7539	51 ± 5 %
10	369-389	AGLLCHLDDACISNPCHK	594.7645	4+	Xyl-Glc	594.0902	13 ± 6 %	594.3344	0 ± 0%	594.6254	48 ± 5 %
10	369-389	AGLLCHLDDACISNPCHK	672.7751	4+	Xyl-Xyl-Glc	672.1874	21 ± 4 %	672.6055	0 ± 0 %	672.1811	0 ± 0 %
12	453-474	CEMDINECHSDPCQNDATCLDK	953.6956	3+	naked	ND	0 ± 0 %	ND	0 ± 0 %	953.3561	0 ± 0 %
12	453-474	CEMDINECHSDPCQNDATCLDK	1007.7132	3+	Glc	ND	0 ± 0 %	1007.7146	82 ± 7 %	ND	0 ± 0 %
12	453-474	CEMDINECHSDPCQNDATCLDK	1051.7273	3+	Xyl-Glc	1051.3447	3 ± 4 %	ND	0 ± 0 %	1051.7280	98 ± 1 %
12	453-474	CEMDINECHSDPCQNDATCLDK	1095.7414	3+	Xyl-Xyl-Glc	1096.0747	97 ± 4 %	1096.0762	2 ± 1 %	ND	0 ± 0 %
12	453-474	CEMDINECHSDPCQNDATCLDK	1061.7308	3+	Hex-Glc	ND	0 ± 0 %	1061.7318	16 ± 6 %	ND	1 ± 1 %
13	488-512	GVHCELEVNECQSNPCVNNGQCVDK	983.0808	3+	naked	983.4023	0 ± 0 %	ND	1 ± 1 %	ND	0 ± 0 %

13	488-512	GVHCELEVNECQSNPCVNNGQCVDK	1037.0984	3+	Glc	ND	0 ± %	1037.4320	97 ± 1 %	ND	0 ± 1 %
13	488-512	GVHCELEVNECQSNPCVNNGQCVDK	1081.1125	3+	Xyl-Glc	1081.6968	4 ± 3 %	ND	0 ± 0 %	1081.4450	99 ± 1 %
13	488-512	GVHCELEVNECQSNPCVNNGQCVDK	1125.1266	3+	Xyl-Xyl-Glc	1125.4593	95 ± 3 %	1125.1272	1 ± 0 %	1125.7937	0 ± 0 %
14	516-546	FQCLCPPGFTGPVCQIDIDDCSSTPCLNGAK	1240.2063.	3+	naked	1241.2601	0 ± 0 %	ND	0 ± 0 %	ND	0 ± 0 %
14	516-546	FQCLCPPGFTGPVCQIDIDDCSSTPCLNGAK	1294.2239	3+	Glc	1294.7448	4 ± 0 %	1294.5574	100 ± 0 %	ND	1 ± 1 %
14	516-546	FQCLCPPGFTGPVCQIDIDDCSSTPCLNGAK	1338.2380	3+	Xyl-Glc	1338.3971	4 ± 2 %	ND	0 ± 0 %	1338.5712	99 ± 1 %
14	516-546	FQCLCPPGFTGPVCQIDIDDCSSTPCLNGAK	1382.2521	3+	Xyl-Xyl-Glc	1382.2915	92 ± 2 %	ND	0 ± 0 %	ND	0 ± 0 %
16	611-619	CYSSPCLND	549.6975	2+	naked	549.3762	1 ± 1 %	549.8763	17 ± 3 %	549.1278	6 ± 0 %
16	611-619	CYSSPCLND	630.7239	2+	Glc	630.2768	9 ± 1 %	630.0487	83 ± 3 %	630.4875	26 ± 4 %
16	611-619	CYSSPCLND	696.7451	2+	Xyl-Glc	696.6941	6 ± 5 %	696.4211	0 ± 0 %	696.7422	68 ± 4 %
16	611-619	CYSSPCLND	762.7662	2+	Xyl-Xyl-Glc	762.8571	84 ± 5 %	762.3674	0 ± 0 %	762.4855	0 ± 0 %
18	679-692	CNIDIDECASNPCR	862.3458	2+	naked	861.3684	3 ± 0 %	862.2873	1 ± 1 %	862.0773	2 ± 1 %
18	679-692	CNIDIDECASNPCR	943.3722	2+	Glc	943.4132	2 ± 0 %	943.5864	98 ± 1 %	943.2765	1 ± 0 %
18	679-692	CNIDIDECASNPCR	1009.3934	2+	Xyl-Glc	1009.3941	37 ± 3 %	1009.9366	0 ± 0 %	1009.4018	97 ± 1 %
18	679-692	CNIDIDECASNPCR	1075.9159	2+	Xyl-Xyl-Glc	1075.9162	59 ± 3 %	1076.8523	0 ± 0 %	1075.3864	0 ± 0 %
19	706-742	CICPEGPHHPSCYSQVNECLSNPCIHGDCTGGLSGYK	850.558	5+	naked	ND	0 ± 0 %	850.1234	9 ± 1 %	ND	0 ± 0 %
19	706-742	CICPEGPHHPSCYSQVNECLSNPCIHGDCTGGLSGYK	882.9686	5+	Glc	ND	0 ± 0 %	882.8974	91 ± 1 %	882.1453	1 ± 0 %
19	706-742	CICPEGPHHPSCYSQVNECLSNPCIHGDCTGGLSGYK	909.3770	5+	Xyl-Glc	ND	0 ± 0 %	ND	0 ± 0 %	909.3866	99 ± 0 %
19	706-742	CICPEGPHHPSCYSQVNECLSNPCIHGDCTGGLSGYK	935.7855	5+	Xyl-Xyl-Glc	935.9877	100 ± 0 %	ND	0 ± 0 %	ND	0 ± 0 %
20	759-780	NECLSNPCQNGGTCNNLVNGYR	896.3798	3+	naked	ND	0 ± 0 %	ND	0 ± 0 %	ND	0 ± 0 %
20	759-780	NECLSNPCQNGGTCNNLVNGYR	950.3947	3+	Glc	ND	0 ± 0 %	950.7438	100 ± 0 %	ND	0 ± 0 %
20	759-780	NECLSNPCQNGGTCNNLVNGYR	994.4115	3+	Xyl-Glc	ND	0 ± 0 %	ND	0 ± 0 %	994.6183	100 ± 0 %
20	759-780	NECLSNPCQNGGTCNNLVNGYR	1038.426	3+	Xyl-Xyl-Glc	1038.7952	100 ± 0 %	ND	0 ± 0 %	ND	0 ± 0 %
21	789-828	GYNCQVNIDECASNPCLNQGTCFDDVSGYTCHCMLPYTGK	1213.9940	4+	naked	1213.4637	0 ± 0 %	1213.3468	0 ± 0 %	1213.4711	0 ± 0 %
21	789-828	GYNCQVNIDECASNPCLNQGTCFDDVSGYTCHCMLPYTGK	1254.5072	4+	Glc	1254.3798	16 ± 4 %	1254.6768	100 ± 0 %	1254.8715	5 ± 3%

21	789-828	GYNCQVNIDECASNPCLNQGTCFDDVSGYTCHCMLPYTGK	1287.5178	4+	Xyl-Glc	1287.0604	5 ± 5 %	1287.1676	0 ± 0 %	1287.4986	95 ± 3 %
21	789-828	GYNCQVNIDECASNPCLNQGTCFDDVSGYTCHCMLPYTGK	1320.5284	4+	Xyl-Xyl-Glc	1320.5296	79 ± 5 %	1320.2934	0 ± 0 %	1319.3785	0 ± 0 %
23	870-880	CTVDVDECISK	663.2895	2+	naked	ND	100 ± 0 %	ND	100 ± 0 %	ND	100 ± 0 %
23	870-880	CTVDVDECISK	744.3159	2+	Glc	ND	0 ± 0 %	ND	0 ± 0 %	ND	0± 0 %
23	870-880	CTVDVDECISK	810.3371	2+	Xyl-Glc	ND	0 ± 0 %	ND	0 ± 0 %	ND	0 ± 0 %
23	870-880	CTVDVDECISK	876.3582	2+	Xyl-Xyl-Glc	ND	0 ± 0 %	ND	0 ± 0 %	ND	0 ± 0 %
25	946-959	CQTDMNECLSEPCK	886.3419	2+	naked	886.4763	3 ± 0%	886.4476	2 ± 1 %	886.9701	0 ± 0 %
25	946-959	CQTDMNECLSEPCK	967.3683	2+	Glc	967.4822	0 ± 0 %	967.2399	98 ± 1 %	967.0544	0 ± 0 %
25	946-959	CQTDMNECLSEPCK	1033.3894	2+	Xyl-Glc	1033.4769	31 ± 5 %	1033.2768	0 ± 0 %	1033.3519	100 ± 0 %
25	946-959	CQTDMNECLSEPCK	1099.9120	2+	Xyl-Xyl-Glc	1099.2865	66 ± 5 %	1099.4700	0 ± 0 %	1100.8355	0 ± 0 %
27	1022-1047	CLHDINECSSNPCLNAGTCVDGLGTY	1019.418	3+	naked	ND	0 ± 0 %	ND	0 ± 0 %	ND	0 ± 0 %
27	1022-1047	CLHDINECSSNPCLNAGTCVDGLGTY	1073.435	3+	Glc	1073.4826	0 ± 0 %	1073.2535	100 ± 0 %	1073.9637	0 ± 0 %
27	1022-1047	CLHDINECSSNPCLNAGTCVDGLGTY	1117.449	3+	Xyl-Glc	1117.3249	0 ± 0 %	1117.1125	0 ± 0 %	1117.2877	100 ± 0 %
27	1022-1047	CLHDINECSSNPCLNAGTCVDGLGTY	1161.463	3+	Xyl-Xyl-Glc	1161.5543	100 ± 0 %	1161.9422	0 ± 0 %	1161.7350	0 ± 0 %
30	1153-1174	CASNPCQHGATCNDFIGGYRCE	880.3401	3+	naked	ND	0 ± 0 %	ND	0 ± 0 %	ND	0 ± 0 %
30	1153-1174	CASNPCQHGATCNDFIGGYRCE	961.3665	3+	Glc	961.5782	6 ± 1 %	961.7420	100 ± 0 %	961.4750	4 ± 1 %
30	1153-1174	CASNPCQHGATCNDFIGGYRCE	1005.3806	3+	Xyl-Glc	1005.6794	31 ± 4 %	1005.3349	0 ± 0 %	1005.3637	96 ± 1 %
30	1153-1174	CASNPCQHGATCNDFIGGYRCE	1049.3947	3+	Xyl-Xyl-Glc	1049.2871	63 ± 4 %	1049.2864	0 ± 0 %	1049.0572	0 ± 0 %
33	1261-1285	CEGDINECLSNPCSSEGSLDCVQLK	958.0696	3+	naked	958.4327	0 ± 0 %	958.2374	0 ± 0 %	958.8433	1 ± 1 %
33	1261-1285	CEGDINECLSNPCSSEGSLDCVQLK	1012.0873	3+	Glc	1012.3769	0 ± 0 %	ND	0 ± 0 %	1012.5469	0 ± 1 %
33	1261-1285	CEGDINECLSNPCSSEGSLDCVQLK	1056.1014	3+	Xyl-Glc	1056.1872	61 ± 3 %	1056.4498	8 ± 1 %	1056.7634	99 ± 1 %
33	1261-1285	CEGDINECLSNPCSSEGSLDCVQLK	1100.1155	3+	Xyl-Xyl-Glc	1100.1866	39 ± 3 %	1100.3799	0 ± 1 %	1100.2871	0 ± 0 %
33	1176-1205	HGSDCSEEINECLSQPCQNGGTCIDLTNSY	1066.1049	3+	Hex-Glc	ND	0 ± 0 %	1066.1059	82 ± 6 %	ND	0 ± 0 %
33	1176-1205	HGSDCSEEINECLSQPCQNGGTCIDLTNSY	1163.1367	3+	Neu5Ac-Hex-Glc	ND	0 ± 0 %	1163.7834	10 ± 6 %	ND	0 ± 0 %

36	1373-1393	DCESGCASNPCQHGGTCYPQR	863.3297	3+	naked	863.6824	2 ± 1 %	863.4876	3 ± 2 %	863.9731	3 ± 1 %
36	1373-1393	DCESGCASNPCQHGGTCYPQR	917.3473	3+	Glc	917.9186	66 ± 10 %	917.2323	97 ± 2 %	917.2111	58 ± 5 %
36	1373-1393	DCESGCASNPCQHGGTCYPQR	961.3614	3+	Xyl-Glc	961.2832	0 ± 1 %	961.7351	0 ± 0 %	961.4577	39 ± 6 %
36	1373-1393	DCESGCASNPCQHGGTCYPQR	1005.3755	3+	Xyl-Xyl-Glc	1005.473 3	30 ± 10 %	1005.832 2	0 ± 0 %	ND	0 ± 0 %

Table S3. Summary of mass spectral semi-quantification of NOTCH1 *O*-fucose glycans. The number of EGF repeats, the number of amino acids counted from the N-terminal, the peptide sequences, predicted mass (m/z), charge state, glycoform, measured mass (m/z), and glycoform ratio of the analyzed peptides are shown. In the glycoform ratio column, the average height of EICs and the standard error of the mean are shown (N=3). Colored letters in sequences indicate post-translational modification sites. Red, *O*-Fuc.

						WT		*GXYLT1/2* DKO		*XXYLT1* KO	
EGF	A.A.	Sequence	Predicted mass (m/z)	charge state	Glycoform	measured mass (m/z)	Glycoform Ratio	measured mass (m/z)	Glycoform Ratio	measured mass (m/z)	Glycoform Ratio
2	113-126	NGG**T**CDLLTLTEYK	792.8829	2+	naked	792.6573	1 ± 0 %	793.0288	1 ± 0 %	792.1849	1 ± 0 %
2	113-126	NGG**T**CDLLTLTEYK	865.9118	2+	Fuc	865.7168	99 ± 0 %	865.9923	99 ± 1 %	866.4625	99 ± 0 %
6	208-234	ATHTGPHCELPYVPCSPSPCQNGG**T**CR	760.8289	4+	naked	760.9859	2 ± 1 %	760.1754	7 ± 2 %	760.4492	5 ± 1 %
6	208-234	ATHTGPHCELPYVPCSPSPCQNGG**T**CR	797.3434	4+	Fuc	797.5814	98 ± 1 %	797.0067	93 ± 2 %	797.5836	95 ± 1 %
36	1369-1413	SPTCLCLGSFTGPECQFPASSPCVGSNPCYNQG**T**CEPTSENPFYR	1371.5691	4+	naked	1371.2829	1 ± 1 %	1371.5901	0 ± 0 %	1371.2299	0 ± 0 %
36	1369-1413	SPTCLCLGSFTGPECQFPASSPCVGSNPCYNQG**T**CEPTSENPFYR	1408.0836	4+	Fuc	1408.3744	99 ± 1 %	1407.6376	100 ± 0 %	1408.1857	100 ± 0 %

Table S4. Summary of mass spectral semi-quantification of NOTCH1 *O*-GlcNAc glycans. The number of EGF repeats, the number of amino acids counted from the N-terminal, the peptide sequences, predicted mass (m/z), charge state, glycoform, measured mass (m/z), and glycoform ratio of the analyzed peptides are shown. In the glycoform ratio column, the average height of EICs and the standard error of the mean are shown (N=3). Colored letters in sequences indicate post-translational modification sites. Blue, *O*-Glc; red, *O*-Fuc; green, *O*-GlcNAc.

						WT		*GXYLT1/2* DKO		*XXYLT1* KO	
EGF	A.A.	Sequence	Predicted mass (m/z)	charge state	Glycoform	measured mass (m/z)	Glycoform Ratio	measured mass (m/z)	Glycoform Ratio	measured mass (m/z)	Glycoform Ratio
2	70-112	NAGTCHVVDHGGTVDYACSCPLGFSGPLCLTPLDNACLANPCR	1213.5366	4+	naked	1213.4516	71 ± 6 %	1213.8911	77 ± 3 %	1213.1087	70 ± 6 %
2	70-112	NAGTCHVVDHGGTVDYACSCPLGFSGPLCLTPLDNACLANPCR	1264.3064	4+	GlcNAc	1264.4866	3 ± 1 %	1264.3458	2 ± 1 %	1264.7924	2 ± 1 %
2	70-112	NAGTCHVVDHGGTVDYACSCPLGFSGPLCLTPLDNACLANPCR	1304.8196	4+	Gal-GlcNAc	1304.5226	9 ± 1 %	1304.9345	6 ± 1 %	1304.0083	5 ± 1 %
2	70-112	NAGTCHVVDHGGTVDYACSCPLGFSGPLCLTPLDNACLANPCR	1377.5935	4+	Neu5Ac-Gal-GlcNAc	1377.4379	17 ± 3 %	1377.7476	14 ± 2 %	1377.9954	23 ± 5 %
10	396-428	AICTCPSGYTGPACSQDVDECALGANPCEHAGK	1185.4912	3+	naked	1185.6047	91 ± 0 %	1185.6248	100 ± 0 %	1185.2671	99 ± 0 %
10	396-428	AICTCPSGYTGPACSQDVDECALGANPCEHAGK	1253.1843	3+	GlcNAc	1253.4771	8 ± 0 %	1253.4558	0 ± 0 %	12541621	0 ± 0 %
10	396-428	AICTCPSGYTGPACSQDVDECALGANPCEHAGK	1307.2019	3+	Gal-GlcNAc	1307.3298	0 ± 0 %	ND	0 ± 0 %	1307.6729	1 ± 0 %
10	396-428	AICTCPSGYTGPACSQDVDECALGANPCEHAGK	1404.2337	3+	Neu5Ac-Gal-GlcNAc	1404.5557	0 ± 0 %	1403.9883	0 ± 0 %	ND	0 ± 0 %
20	781-814	EGFSGPNCQTNINECASNPCLNQGTCIDDVAGYK	1091.7014	4+	naked	1091.8113	17 ± 1 %	1091.4195	17 ± 3 %	1092.0195	24 ± 9 %
20	781-814	EGFSGPNCQTNINECASNPCLNQGTCIDDVAGYK	1142.4713	4+	GlcNAc	1142.7856	2 ± 1 %	1142.9897	10 ± 1 %	1142.8822	9 ± 6 %
20	781-814	EGFSGPNCQTNINECASNPCLNQGTCIDDVAGYK	1182.9845	4+	Gal-GlcNAc	1183.0492	64 ± 2 %	1182.3814	58 ± 7 %	1182.3569	51 ± 5 %
20	781-814	EGFSGPNCQTNINECASNPCLNQGTCIDDVAGYK	1255.7584	4+	Neu5Ac-Gal-GlcNAc	1255.3632	16 ± 1 %	1255.6902	15 ± 4 %	1255.4821	16 ± 2 %
